# Supplementary material for: Investigation of the impacts of climate change and rising temperature on food poisoning cases in Malaysia
Source: PLoS One. 2023 Oct 20;18(10):e0283133. doi: 10.1371/journal.pone.0283133 (PMC10588843; doi:10.1371/journal.pone.0283133)
Supplement: Attachment — Submitted filename: PONE-D-21-31372_R1_reviewer (1).pdf [file pone.0283133.s001.pdf]

PLOS ONE

Climate Change and Temperature Rise: Implications on Food Poisoning Cases in Malaysia

--Manuscript Draft--

**Manuscript Number:**	PONE-D-21-31372R1
**Article Type:**	Research Article
**Full Title:**	Climate Change and Temperature Rise: Implications on Food Poisoning Cases in Malaysia
**Short Title:**	Climate Change and Temperature Rise: Implications on Food Poisoning Cases in Malaysia
**Corresponding Author:**	Noor Artika Hassan, PhD<br>International Islamic University Malaysia<br>Kuantan, MALAYSIA
**Keywords:**	Climate change, temperature, food poisoning, Malaysia, rainfall
**Abstract:**	Background/Aim : This study is an attempt to quantify climate-induced increases in morbidity rates of food poisoning cases.<br>Methods: Monthly food poisoning cases, average monthly meteorological data, and population data from 2004 to 2014 were obtained from the Malaysian Ministry of Health, Malaysian Meteorological Department, and Department of Statistics Malaysia, respectively. Poisson generalized linear models were developed to quantify the association between climatic parameters and the number of reported food poisoning cases.<br>Results : The findings revealed that the food poisoning incidence proportion in Malaysia during the 11 years study period was 561 cases per 100 000 population for the whole country. Among the cases, females and the ethnic Malays most frequently experienced food poisoning with incidence proportion of 313 cases per 100,000 and 438 cases per 100,000 population over the period of 11 years, respectively. Most of the cases occurred within the active age of 13 to 35 years old. Temperature gave a significant impact on the incidence of food poisoning cases in Selangor (p=0.020), Melaka (p=0.027), Kelantan (p=0.005), and Sabah (p=0.012) while rainfall was a protective factor in Terengganu (p=0.034). For a 1.0 °C increase in temperature, the excess risk of food poisoning in each state can increase up to 74.1% whereas for every 50mm increase in rainfall, the risk of getting food poisoning is decreased by almost 10%.<br>Conclusion: The study concludes that climate does affect the distribution of food poisoning cases in Selangor, Melaka, Kelantan, Sabah, and Terengganu. Food poisoning cases in other states are not directly associated with temperature but related to monthly trends and seasonality.
**Order of Authors:**	Noor Artika Hassan, PhD
	Jamal Hisham Hashim, PhD
	Sharifa Ezat Wan Puteh
	Wan Rozita Wan Mahiyuddin
	Mohd Syazwan Faisal Mohd
	Shazlyn Milleana Shaharudin
	Edre Mohammad Aidid
	Isnizam Sapuan
**Response to Reviewers:**	Dear editor,<br><br>Previously, the paper was rejected due to no reviewers to review the paper. I have suggested 3 names which are as follows :<br><br>1) Dr Sao Vibol, vibol.sao@rupp.edu.kh, from Cambodia and

Powered by Editorial Manager® and ProduXion Manager® from Aries Systems Corporation

	2) Prof Budi Haryanto from Indonesia, bharyanto@ui.ac.id<br><br>3)Associate Prof Dr Rozita Hod, rozita.hod@ppukm.ukm.edu.my from Malaysia
**Additional Information:**	
**Question**	**Response**
**Financial Disclosure**<br><br>Enter a financial disclosure statement that describes the sources of funding for the work included in this submission. Review the submission guidelines for detailed requirements. View published research articles from *PLOS ONE* for specific examples.<br><br>This statement is required for submission and **will appear in the published article** if the submission is accepted. Please make sure it is accurate.<br><br>**Unfunded studies**<br>Enter: *The author(s) received no specific funding for this work.*<br><br>**Funded studies**<br>Enter a statement with the following details:<br>• Initials of the authors who received each award<br>• Grant numbers awarded to each author<br>• The full name of each funder<br>• URL of each funder website<br>• Did the sponsors or funders play any role in the study design, data collection and analysis, decision to publish, or preparation of the manuscript?<br>• **NO** - Include this sentence at the end of your statement: *The funders had no role in study design, data collection and analysis, decision to publish, or preparation of the manuscript.*<br>• **YES** - Specify the role(s) played.<br><br>* typeset	This research received financial assistance from IRAGS18-048-0049.
**Competing Interests**<br><br>Use the instructions below to enter a competing interest statement for this submission. On behalf of all authors, disclose any competing interests that could be perceived to bias this work—acknowledging all financial support	The authors have declared that no competing interests exist.

and any other relevant financial or non-financial competing interests.<br><br>This statement is required for submission and **will appear in the published article** if the submission is accepted. Please make sure it is accurate and that any funding sources listed in your Funding Information later in the submission form are also declared in your Financial Disclosure statement.<br><br>View published research articles from *PLOS ONE* for specific examples.<br><br>**NO authors have competing interests**<br><br>Enter: *The authors have declared that no competing interests exist.*<br><br>**Authors with competing interests**<br><br>Enter competing interest details beginning with this statement:<br><br>*I have read the journal's policy and the authors of this manuscript have the following competing interests: [insert competing interests here]*<br><br>* typeset	
**Ethics Statement**<br><br>Enter an ethics statement for this submission. This statement is required if the study involved:<br><br>• Human participants<br>• Human specimens or tissue<br>• Vertebrate animals or cephalopods<br>• Vertebrate embryos or tissues<br>• Field research<br><br>Write "N/A" if the submission does not require an ethics statement.<br><br>General guidance is provided below.	Ethical approval has been obtained from the Faculty Medicine Universiti Kebangsaan Malaysia (FF-2013-424) and National Medical Research Register (NMRR-13-1120-17533).

Powered by Editorial Manager® and ProduXion Manager® from Aries Systems Corporation

Consult the submission guidelines for detailed instructions. **Make sure that all information entered here is included in the Methods section of the manuscript.**<br><br>**Format for specific study types**<br><br>**Human Subject Research (involving human participants and/or tissue)**<br>• Give the name of the institutional review board or ethics committee that approved the study<br>• Include the approval number and/or a statement indicating approval of this research<br>• Indicate the form of consent obtained (written/oral) or the reason that consent was not obtained (e.g. the data were analyzed anonymously)<br><br>**Animal Research (involving vertebrate animals, embryos or tissues)**<br>• Provide the name of the Institutional Animal Care and Use Committee (IACUC) or other relevant ethics board that reviewed the study protocol, and indicate whether they approved this research or granted a formal waiver of ethical approval<br>• Include an approval number if one was obtained<br>• If the study involved *non-human primates*, add *additional details* about animal welfare and steps taken to ameliorate suffering<br>• If anesthesia, euthanasia, or any kind of animal sacrifice is part of the study, include briefly which substances and/or methods were applied<br><br>**Field Research**<br><br>Include the following details if this study involves the collection of plant, animal, or other materials from a natural setting:<br>• Field permit number<br>• Name of the institution or relevant body that granted permission	
**Data Availability**<br><br>Authors are required to make all data underlying the findings described fully available, without restriction, and from the	No - some restrictions will apply

Powered by Editorial Manager® and ProduXion Manager® from Aries Systems Corporation

time of publication. PLOS allows rare exceptions to address legal and ethical concerns. See the PLOS Data Policy and FAQ for detailed information.<br><br>A Data Availability Statement describing where the data can be found is required at submission. Your answers to this question constitute the Data Availability Statement and **will be published in the article**, if accepted.<br><br>**Important:** Stating 'data available on request from the author' is not sufficient. If your data are only available upon request, select 'No' for the first question and explain your exceptional situation in the text box.<br><br>Do the authors confirm that all data underlying the findings described in their manuscript are fully available without restriction?	
**Describe where the data may be found in full sentences. If you are copying our sample text, replace any instances of XXX with the appropriate details.**<br><br>• If the data are **held or will be held in a public repository**, include URLs, accession numbers or DOIs. If this information will only be available after acceptance, indicate this by ticking the box below. For example: *All XXX files are available from the XXX database (accession number(s) XXX, XXX.*).<br>• If the data are all contained **within the manuscript and/or Supporting Information files**, enter the following: *All relevant data are within the manuscript and its Supporting Information files.*<br>• If neither of these applies but you are able to provide **details of access elsewhere**, with or without limitations, please do so. For example:<br><br>*Data cannot be shared publicly because of [XXX]. Data are available from the XXX Institutional Data Access / Ethics Committee (contact via XXX) for*	All the raw morbidity data from Malaysia are confidential as it contain personal information such as ID, address and other personal information. However, the climate data can be shared to all readers.

researchers who meet the criteria for access to confidential data.<br><br>*The data underlying the results presented in the study are available from (include the name of the third party and contact information or URL).*<br>• This text is appropriate if the data are owned by a third party and authors do not have permission to share the data.<br><br>* typeset	
Additional data availability information:	Tick here if your circumstances are not covered by the questions above and you need the journal's help to make your data available.

Powered by Editorial Manager® and ProduXion Manager® from Aries Systems Corporation

Manuscript Click here to access/download;Manuscript;Manuscript.docx

Climate Change and Temperature Rise: Implications on Food Poisoning Cases in Malaysia

Noor Artika Hassan [a,b,c] , Jamal Hisham Hashim [b,c,d] , Sharifa Ezat Wan Puteh [b] , Wan Rozita Wan Mahiyuddin [e] , Mohd Syazwan Faisal Mohd [f] , Shazlyn Milleana Shaharudin [g] , Edre Mohammad Aidid [a], Isnizam Sapuan [a]

[a] Department of Community Medicine, Kulliyyah of Medicine, International Islamic University Malaysia, 25200 Kuantan, Pahang, Malaysia

[b] Department of Community Health, Faculty of Medicine, UKM Medical Centre, Bandar Tun Razak, 56000 Kuala Lumpur, Malaysia

[c] United Nations University-International Institute for Global Health, UKM Medical Centre, Bandar Tun Razak, 56000 Kuala Lumpur, Malaysia

[d] Department of Health Sciences, Universiti Selangor, 40000 Shah Alam, Selangor, Malaysia

[e] Institute for Medical Research, Ministry of Health, 50588 Kuala Lumpur, Malaysia

[f] National Hydraulic Research Institute of Malaysia, Ministry of Environment , 43300 Seri Kembangan, Selangor, Malaysia

[g] Department of Mathematics, Faculty of Science and Mathematics, Universiti Pendidikan Sultan Idris, Malaysia

Background/Aim : This study is an attempt to quantify climate-induced increases in morbidity rates of food poisoning cases.

Methods: Monthly food poisoning cases, average monthly meteorological data, and population data from 2004 to 2014 were obtained from the Malaysian Ministry of Health, Malaysian Meteorological Department, and Department of Statistics Malaysia, respectively. Poisson generalized linear models were developed to quantify the association between climatic parameters and the number of reported food poisoning cases.

Results : The findings revealed that the food poisoning incidence proportion in Malaysia during the 11 years study period was 561 cases per 100 000 population for the whole country. Among the cases, females and the ethnic Malays most frequently experienced food poisoning with incidence proportion of 313 cases per 100,000 and 438 cases per 100,000 population over the period of 11 years, respectively. Most of the cases occurred within the active age of 13 to 35 years old. Temperature gave a significant impact on the incidence of food poisoning cases in Selangor (p=0.020), Melaka (p=0.027), Kelantan (p=0.005), and Sabah (p=0.012) while rainfall was a protective factor in Terengganu (p=0.034). For a 1.0 °C increase in temperature, the excess risk of food poisoning in each state can increase up to 74.1% whereas for every 50mm increase in rainfall, the risk of getting food poisoning is decreased by almost 10%.

Conclusion: The study concludes that climate does affect the distribution of food poisoning cases in Selangor, Melaka, Kelantan, Sabah, and Terengganu. Food poisoning cases in other states are not directly associated with temperature but related to monthly trends and seasonality.

Corresponding author : Noor Artika Hassan, noor_artika@iium.edu.my

Introduction

Food poisoning is defined as an acute onset of vomiting, diarrhoea, or other acute symptoms associated with the ingestion of compromised food or drinks (MOH, 2017). It can also cause neurological symptoms such as muscle weakness, paraesthesia, paralysis, and even death. It is listed as one of the notifiable diseases and require mandatory notification under the Prevention and Control of Infectious Disease Act 1988 in Malaysia. Food poisoning is usually infectious or toxic and the causal agents can be pathogenic microbes, including bacteria (e.g. *Salmonella*, *E. coli*, and *Campylobacter*), viruses (e.g. Norovirus), parasites (e.g. *Ascaris, Cryptosporidium,*

Entamoeba histolytica or *Giardia*), chemicals (e.g. mycotoxins, marine biotoxins, persistent organic pollutants, heavy metals) or fungi (e.g. *Aspergillus*). An outbreak of food poisoning is declared if there are two or more cases of a similar illness resulting from the ingestion of the same contaminated food or drink (WHO, 2017). Foods that are most commonly contaminated in outbreaks are raw or undercooked poultry, dairy products, expired eggs, and vegetables and mostly from *Salmonella Typhi, Staphylococcus aureus, Escherichia coli* and *Clostridium perfringens* (Abdul Mutalib *et al.*, 2015). Various factors may contribute to food poisoning like poor self-hygiene of food handlers, unavailability of clean water supply and unhygienic kitchen utensils or kitchen counters (Abdul Mutalib, 2015).

Food poisoning incidences in Malaysia showed an increasing trend over the past twenty years (Meftahudin, 2002; MOH, 2006). In the 1980s, the incidences were below 10.2 cases per 100,000 population, but the incidences increased to more than 35.0 cases per 100,000 population, and consistently above 44.0 cases per 100,000 population in 1999 and after 2010, respectively (Meftahudin, 2002; MOH, 2006; Abdul Mutalib *et al.*, 2015). The highest incidence recorded at the state level was in Melaka in 1996, with the incidence of 197.05 cases per 100,000 population (Meftahudin, 2002), followed by 58.91 cases per 100,000 population in Kelantan in 2005 (MOH, 2006).

The distribution and incidence of food poisoning are influenced by fluctuations in weather patterns such as temperature, rainfall, and relative humidity as a result of climate change (El-fadel M. *et al*., 2012; Ghazani *et al*., 2018; Liu *et al.*, 2019; Wang 2018). In countries that have a seasonal temperate climate, it was proven that food poisoning illnesses peak during warmer months (El-fadel M. *et al*., 2012). However, data from tropical countries like Malaysia where it is hot and humid throughout the year are limited to support the correlation of high food poisoning incidences in perpetually hot climate regions. Climate in

Malaysia is influenced by the mountainous topography and complex land-sea interactions. The interannual variability of temperature in Malaysia is primarily dominated by the El Nino-Southern Oscillation (ENSO). Warm and dry climate provides favorable growth of pathogenic microbes, including those causing food-borne illnesses in the food supply chain (Woodward *et al*.2014; El-fadel M. *et al*., 2012; Ghazali *et al*., 2018). A warm climate can also affect human behaviours on food consumption and social interaction. People tend to drink more water, get more involved with recreational activities, and attend social gatherings like a wedding during the Southwest Monsoon (warmer months). These conditions increase the risk of food poisoning due to improper management in raw food supply, preparation, serving or during storage.

Based on the Representative Concentration Pathways (RCPs) emissions, the scenarios indicated that the minimum increase of global mean surface temperature is likely to be from 0.3 °C to 1.7 °C under RCP 2.6, while the maximum increase is likely to be from 2.6 °C to 4.8°C under RCP 8.5 at the end of the 21st century (IPCC 2014). In Malaysia, the observed surface temperature data had also indicated an increase in warming between 0.32 and 1.5 °C for the last five decades (MMD, 2009; Wong *et al*.,2018). Peninsular Malaysia indicated a higher temperature increase compared to East Malaysia, and western Peninsular Malaysia indicated the highest compared to other states (MMD, 2009). Malaysia also receives abundant rainfall annually and the East Coast areas experienced floods almost every year during the Northeast Monsoon (rainy season). With the increasing temperature, abundant rainfall during the Northeast Monsoon, and elevated food poisoning incidences, research on climatic factors influencing food poisoning incidences in hot tropical countries are essential. Previous research used Generalized linear Model (GLMs) (McIver, L *et al.*, 2015; Hassan *et al*., 2020; Onozuka and Hashizume, 2011), Generalized Additive Model (GAM) (Lin *et al.*, 2016), Quasi-Poisson regression (Zhou *et al*., 2013), and Pearson correlation regression (Kim, 2020) to examine the association between climate variables and infectious diseases, however GLMs are the most

widely used. Therefore, this research aims to investigate the influence of climate variability on food poisoning cases in Malaysia using a Poisson generalized linear model. This research is important for future prediction of food poisoning in extreme climatic scenarios and taking into consideration the appropriate intervention measures that could be done in Malaysia.

Methodology

Study population

The study included the Malaysian population, irrespective of race, in all 13 states, including Sabah and Sarawak. The total population in Malaysia in 2014 was 30.72 million. Native Malays or Bumiputera recorded the highest number with 18.87 million (61.4%), followed by Chinese and Indians with 6.59 million (21.5%) and 1.98 million (6.4%), respectively. Non-Malaysians were at 3.01 million (9.8%) while others were 0.27 million (0.9%) (DOSM, 2016).

Food poisoning data, population data, and climate data

In order to explore the relationship between climate and food poisoning cases in Malaysia, the monthly data of food poisoning cases were collected from January 2004 to December 2014 from the Ministry of Health (MOH). For demographic data, basic information such as the locality or address, age, gender, race, date of onset, and date of analysis were recorded. All incomplete dataset without medical information such as the onset date, sampling date, and diagnosis date were rejected. All these food poisoning cases were converted to incidence data by dividing it with the population data obtained from the Department of Statistics Malaysia (DOSM).

Monthly climate data such as relative humidity, temperature, and rainfall were obtained from MMD for the years 2004 to 2014. The missing data were estimated using the multiple regression analysis (multiple imputation method) in SPSS. The collinearity between independent variables for each state was analysed prior to model development using the correlation test and Variance Inflation Factors (VIF). Data were analysed using IBM SPSS for Windows version 23. Descriptive analysis was used to describe the demographic data, meteorological data, and morbidity data. A generalized linear model was applied for multivariate analyses.

The mean and maximum data for incidence of food poisoning, temperature and rainfall for the whole Malaysia were mapped using QGIS version 3.4 long-term release. The incidence of food poisoning used choropleth maps while rainfall used the inverse distance weighing (IDW) interpolation method. The classification used the quartile method from Q1 (the lowest) to Q4 (the highest).

Model Development

Poisson generalized linear models and overdispersed Poisson models estimating the understated standard errors were run to investigate the association between climate parameters and food poisoning incidence at lag 0 month and lag 1 month. The model equation for overdispersed Poisson was the same as that for Poisson regression. The overdispersion parameter did not affect the expected outcome, but it affected the estimated variance of the expected counts. Scale weight was used to control for overdispersion by dividing the deviance to 1. Fourier terms and time stratified model were included in the model to control for seasonality and the monthly trends, respectively. Seasonality refers to the presence of variations that occur at regular monthly intervals due to the weather factor. Trend refers to the direction of data that is either increasing or decreasing, and linear assumption was used in this

research. The analysis was considered towards pooled health impacts for the community at the state level. As the population increased over time, the population was set up as an offset.

The framework models were explained in Hassan *et al.* (2020) and El-fadel *et al.* (2012). The framework was accommodated as expressed in Equation (1) to (3):

$$Yi \sim Pois(u_i\lambda_i) \quad (1)$$

$$\log(u_i\lambda_i) = \log(u_i) + \log(\lambda i) \quad (2)$$

$$\log(\lambda i) = Xi\beta \quad (3)$$

Where ui is the exposed population at that time; λi is the expected number of cases as a fraction of the exposed population; Xi is the regression matrix; and β is the vector of model coefficients.

The robustness of the model depends on the goodness of fit. The smallest deviance, Pearson Chi Square, and Akaike information criterion (AIC) were chosen as the best model. Sensitivity testing was conducted to determine the sensitivity of significant independent variables such as temperature and rainfall in affecting food poisoning incidences. We used lag temperature or rainfall data to replace the climate variables (temperature or rainfall) at the Lag 0 data due to the unavailability of minimum and maximum climate data in this research. If the analysis is to ensure consistency of temperature in affecting food poisoning incidences, the lag rainfall data will be used and vice versa. Similar findings with the real model indicated the model was robust.

Results

Food poisoning cases in Malaysia

Table 1 and 2 present the demographic information and annual incidence rate for food poisoning in Malaysia. The annual incidence rate by states ranged from 2.8 to 172.3 cases per 100,000 population while the total incidence proportion of food poisoning for the whole country in Malaysia during the 11-year study period was 561 cases per 100, 000 population. Generally, females and the ethnic Malays most frequently experienced food poisoning with incidence proportion of 313 cases per 100, 000 and 438 cases per 100,000 population over the period of 11 years, respectively. Most of the cases occurred within the active age of 13 to 35 years old. There were some missing data in the demographic information such as race and age. However, the data was not discarded as the main outcome in this research is the total number of population cases per month instead of individual information.

Large variations were found between the minimum, mean, and maximum incidence rates in each state as shown in Figure 1 and 2. Region-wise comparative data showed that the highest annual incidence rate was recorded in Perlis state, while the minimum annual incidence rate was in Selangor state with the incidence rate of 172 cases per 100,000 population and 3 cases per 100,000 population, respectively. High incidence rate refers to the incidence rate above the 75th percentile, while low incidence rate refers to the incidence rate below the 25th percentile. Kelantan and Terengganu states were among the consistently highest incidence rates with the mean annual incidence rate of above 90 cases per 100,000 population. In the southern and central areas of Malaysia, Melaka consistently reported high incidence rates throughout the study period with more than 60 cases per 100,000 population. In the northern region, the incidence rates were high in Penang, Perak, and Perlis with more than 40 cases per 100,000 population. Most of the cases were recorded during the warmer months from April to August. However, states in the northern regions such as Kedah, Penang, Perak, and Perlis, had different trends compared to other states where there were two peak periods which were at the beginning of the year in January and February, and from September and October. The peak periods were

at the end of the northeast monsoon and early intermonsoon in Malaysia, respectively. All the states in Malaysia recorded the least food poisoning incidence rates during the northeast monsoon months in November and December.

Climate variations in Malaysia

Data for the 11-year study period showed that the mean monthly temperature ranged from 24.7 °C to 30.0 °C (Figure 3). The data showed very small temperature variations as Malaysia only has southwest monsoon, northeast monsoon, and inter-monsoon between these two seasons. The maximum values of the mean monthly temperature attained from April to June coinciding with the inter-monsoon and southwest monsoon period with 50.0% of the recorded temperatures were 29.0 °C and above while the minimum values occurred in December or January in conjunction with the northeast monsoon period with 60% of the temperatures being below 25.0 °C (Table 2 and Figure 4). The trend test showed an upward trend of mean monthly temperature for almost all states from 2004–2014. However, the mean monthly temperature increases were small, below 0.5 °C in 2010–2014 compared to 2004–2009 ($p>0.05$).

The rainfall data varied during the study period (Figure 5-6). The mean monthly rainfall and the maximum monthly rainfall for all states were between 150mm/month and 300mm/month, and 350mm/month and 1250mm/month, respectively. High rainfall was recorded during the intermonsoon (October), and northeast monsoon, except for the northern region, whereby the trends differ as the region received high rainfall in March, and August to December. The East Coast areas of Malaysia had rainfall of more than 1000mm/month in November and December. Low rainfall was recorded at the end of the northeast monsoon and southwest monsoon for all states. The months which contributed the high rainfall tally with those months with low temperature as the data showed that temperature and rainfall were

inversely correlated for almost all states (r= −0.713 to −0.296). The trend test showed an upward trend of rainfall for most states in 2004–2014 although the inter-annual changes were found to be insignificant (p>0.05).

Association between climate and food poisoning incidence

Exploratory data analysis revealed a high level of multicollinearity between rainfall and relative humidity. As such, only one of these two factors can be used to ensure model stability. Due to limited information on relative humidity research related to food poisoning compared to rainfall and the abundant rainfall in Malaysia especially during the Northeast monsoon, relative humidity was excluded from the analysis.

The Poisson GLMs and overdispersed Poisson analyses were run for all states and the results showed that temperature was significantly related to food poisoning incidences in Selangor (p=0.020), Kelantan (p=0.005), Melaka (p=0.027), and Sabah (p=0.012) at lag 0 (Table 3 and Figure 7 to 10), and Kelantan (p=0.030) at lag 1 month, while rainfall was significantly related to food poisoning incidences in Terengganu at lag 0 (p=0.034) (Figure 11).

All food poisoning incidences in other states were associated only with seasonality and/or trend. The seasonality indicated that food poisoning rates were elevated mostly during the southwest monsoon and decreased during the northeast monsoon. The overall trend of food poisoning incidences for all states were slightly decreased over the 11 years of study period except for Negeri Sembilan and Perlis. For every 1.0 °C increase in temperature, the excess risks of food poisoning in all states increased between 23.6% and 74.1%. The temperature was also tested at every increase of 0.25 °C, 0.5 °C and 0.75 °C. The results showed an upward increment of excess risks of food poisoning as the temperature increases (Table 4). Rainfall was a protective factor in Terengganu. For every 1mm increase in rainfall, the risk of getting

food poisoning was decreased by less than 1%. Besides 1mm, rainfall was also tested for every increase of 10mm and 50mm and the results showed, the risk can be decreased by almost 10% if the amount of rainfall was increased. The results were consistent with Table 2 as it indicated the high incidence of food poisoning occurred during the southwest monsoon (warmer months) and low incidence in the northeast monsoon (rainy seasons). The deviance after controlling for over-dispersion was 1, and the Akaike's Information Criterion (AIC) was below 150 for all states. The final models were tested for sensitivity analysis, and the results indicated that the temperature and rainfall factors were still significant in Selangor, Kelantan, Melaka and Sabah, even if one of the independent variables was replaced.

Discussion

Many papers discussed the impacts of climate change on diarrhoeal diseases in general but only limited papers were specifically on food poisoning (Bentham, & Langford 1995; D'Souza *et al*. 2004; El-fadel *et al*. 2012; Kendrovski, & Gjorgjev 2012; Kovats *et al*. 2004a). None of this research has been conducted in Southeast Asia, and therefore, the findings from this research will be useful as the baseline for Southeast Asia and tropical climate settings.

The total food poisoning annual incidences in all states in Malaysia fluctuated and ranged from 3 to 172.0 cases per 100,000 population. The variations in the incidences were large, which is due to the denominator difference. Although Selangor was reported as the state with the highest food poisoning cases, the total population in Selangor was the highest in Malaysia, therefore lowering the total incidence cases. The highest annual and monthly incidences were reported in the East Coast states such as Terengganu and Kelantan as it can reach up to 53.0 cases per 100,000 population in a month. Smaller states like Perlis and Melaka also indicated higher incidences due to low population number.

Although all states were affected, only states that have a significant relationship with the climate: Selangor, Kelantan, Melaka, Sabah, and Terengganu, were explained in detail. Temperature alone gave a total percentage of more than 10.0% variation for food poisoning cases in Malaysia. The increase of 1°C of temperature is associated with an excess risk of food poisoning in each state of between 23.6% to 74.1%, and 34.6% for Lag 0 and Lag 1 month, respectively. The risk of getting food poisoning was increased as the temperature increases, as presented in Table 5. Temperature in Kelantan was the only significant variable associated with an increased risk of food poisoning at Lag 1. This situation might be due to the acute effects and a short incubation period of food poisoning, and therefore, all other states showed insignificant findings. Most research showed lower excess risks compared to this research as the increase of 1.0 °C of temperature led to the percentage change of food poisoning cases of between 3.0% to 18.0% (Cullen, 2009; El-fadel *et al.*, 2012; Kendrovski, & Gjorgjev, 2012). Malaysia's climate is hot and humid throughout the year compared to other studies that have four seasons. Most of the food poisoning cases investigated in other studies were related to cases of Salmonellosis food poisoning, while cases in Malaysia are cumulative cases of all causes of food poisoning. Variations in geography, culture, eating behaviour and technology, food type, and hygiene are also essential factors that can trigger food poisoning for example in Malaysia, the least cases were found among Chinese due to the preference to serve food hot from the wok (Abdul Mutalib *et al.*, 2015; Sharifa Ezat *et al.*, 2013; Salleh *et al.*, 2017; Zin *et al.*, 2015; Soon *et al.*,2011). Due to limited research specifically on the role of climatic variations on food poisoning, this research uses diarrhoeal research as the comparison. Zhou *et al.* (2013) and Chou *et al.* (2010) indicated a higher excess risk of getting food poisoning of 80.0% and between 20.0% to 52.0% depending on the age groups, respectively. Different studies recorded different excess risks and relative risks due to differences in terms of geographical area, climate variability, interpretation of enteric infections, method of analysis,

and varying levels of temperature increase as some studies used an increase of 1.0, 2.0, and 5.0 °C (El fadel *et al.*, 2012; Chou *et al*., 2010; Ghazani *et al.*, 2018; Zhou *et al*., 2013).

The research also found that rainfall was only significantly related to food poisoning in Terengganu with a risk reduction of almost 10% for every 50mm increase of rainfall. Several studies supported this inverse relationship, but for diarrhoeal diseases (Beyene *et al.*, 2019; Carlton *et al*., 2014; Morral *et al.*, 2018). Comparing the thresholds with other research was difficult because every area has different climates, rainfall intensities, cut-off points, and time-lags. Terengganu is one of the east coast states that receives abundant rainfall every year during the northeast monsoon season (November–December). During rainy days, people tend to stay at home and cook their own food. The food is usually consumed while it is still hot, therefore lessens the risk of getting food poisoning as there is control of the bacterial growth. In addition, rainfall could also protect the communities against exposure to contaminated recreational water bodies. All other states showed insignificant results which may be due to the monthly mean rainfall that were low in other areas. Rainfall is usually related to floods, and floods are very common in Malaysia. However, based on a literature search conducted, very limited cases of food poisoning were reported during flood events in Malaysia. Badrul *et al*. (2009) reported that only 46 cases of food poisoning occurred during the major floods in Johor in December 2006 and January 2007 whereas during the major flood in 2014, there were only 27 food poisoning cases reported in Pahang (Othman, 2015), and no case was reported in Kelantan (Fauziah, 2015). Minimal cases of food poisoning during flood might be due to the complexity in specifically identifying food poisoning events from other enteric infections including gastroenteritis, or the successful public health interventions such as health education activities in the flood evacuation centres and through the media, improved sanitation and hygiene through the provision of safe water supply and toilets, and efficient management of the disposal of flood wastes and carcasses to prevent disease-breeding grounds.

Between February and July every year, many events, weddings, and family gatherings are usually organized during Chinese New Year, one-week school holidays in March, and the two-week mid-term holidays in May. The increased frequency of these events may elevate the potential of getting food poisoning, especially when the food is not prepared properly (MOH, 2016; Malay Mail, 2020). During the end of the northeast monsoon and southwest monsoon seasons, Malaysia receives a minimum amount of rainfall and therefore, the temperature was high during that period. Most of the food poisoning incidences were reported during the same period. Hot weather provides an optimal temperature range for the growth of many types of toxin-producing bacteria, such as *Staphylococcus aureus* (37.0 °C) (Valík, 2012), *Bacillus cereus* (30.0 °C to 37.0 °C) (ESR, 2015), and *Salmonella* (35 °C to 37 °C) (Health, 2001). These temperatures are within the maximum temperature range in Malaysia. Therefore, bacterial growth and toxin production are easily induced in foods that are not served at a sufficiently high temperature. The low incidence rates of food poisoning in November and December for all states may be related to comparatively low temperature and high rainfall.

The food poisoning incidences in Selangor, Melaka, Kelantan, Terengganu, and Sabah can be due to multiple factors. Selangor and Melaka received the highest temperature distributions after the northern areas. Selangor is also the most populous state in Malaysia, has a high number of squatters in pockets of the urban metropolitan area (Md. Wahid Murad *et al*., 2014) and has unhygienic environment and lack of basic sanitation facilities. In addition, most of the communities in these areas are working and eating outside in the restaurants. People can easily get infections through foods either during preparation, storage or serving (Abdul-Mutalib *et al*., 2015; Sharifa Ezat *et al*., 2013; Soon *et al*., 2011) and the temperature can accelerate the food poisoning infection in these areas. The situation in Melaka is similar to Selangor where food contaminations most possibly occurred in food premises.

Food poisoning incidences in Terengganu, Kelantan and Sabah might have different possible contributing factors. Terengganu received the most abundant rainfall in Malaysia, followed by Kelantan and Pahang. However, rainfall was a protective factor for food poisoning only in Terengganu. In Kelantan and Sabah, high incidences of food poisoning were during the southwest monsoon due to hot weather. Apart from the climatic influences (temperature and rainfall), the large fraction of unexplained variance in food poisoning events observed in this area could be related to the prevalent disparity in the social factor.

In conclusion, climate does affect the distribution of food poisoning cases in Selangor, Melaka, Kelantan, Sabah, and Terengganu. For a 1.0 °C increase in temperature, the excess risk of food poisoning in each state can increase up to 74.1%, whereas for every 50mm increase in rainfall, the risk of getting food poisoning is decreased by almost 10%. The excess risk is directly associated with the hot and humid climate in Selangor, Melaka, Kelantan, and Sabah, and inversely associated with rainfall in Terengganu. High rainfall in Terengganu is a protective factor towards food poisoning. Food poisoning cases in other states are not directly associated with temperature but related to seasonality and/or trends. Different states might have additional different factors that contributed to the food poisoning. Besides the climate factors, different states have other multifactorial issues such as hygiene, sanitation, low coverage of clean water supply, food contamination in the premises, and prevalent disparity in the social factor. In the future, based on the RCP model worldwide, all countries will have at least a minimum increase in temperature from 0.3 °C to 1.7 °C, and Malaysia is not excluded. It will provide an optimum temperature for a lot of pathogenic organisms. Therefore, multidisciplinary approaches are needed to control food poisoning outbreaks and improve food safety in Malaysia. Some of the measures include strengthening the management of water treatment, creating more awareness on food hygiene and sanitation, inculcating the practices of hand washing with soap, and boiling water for at least one minute to kill the microbes (EPA,

2015). It is also advised to cook food at temperatures of more than 70.0 °C to kill all the dangerous microorganisms, and store foods at the safe temperature of below 4°C. To improve food safety, several ministries should strengthen the collaborative works, for example, Ministry of Health, Department of Veterinary, and Department of Fisheries to provide certificates for the imported and exported foods, and strengthen the enforcement related to Food Act 1983. More inspection, food sampling, research activities and quality assurance programs are needed to make food safety in Malaysia successful.

Ethical Approval

Ethical approval has been obtained from the Faculty Medicine Universiti Kebangsaan Malaysia (FF-2013-424) and National Medical Research Register (NMRR-13-1120-17533). This research received financial assistance from IRAGS18-048-0049.

References

1. Abdul Mutalib, N. A., Syafinaz, A. N., Sakai, K. & Shirai, Y. (2015). An overview of foodborne illness and food safety in Malaysia. *International Food Research Journal* 22(3): 896–901.

2. Badrul H, Nor Azian S, Marzuki, Norli, Fatimah, Kee, Arbaiah, MohdYusof, Adam.2009. Spectrum of Flood-related diseases encountered during flood disaster in Johore, Malaysia. *Community Health Journal* 15: 15-23.

3. Beyene, H. (2019). Climate variability associate with childhood diarreha in southern Ethiopia. In *30th EPHA Annual Conference*.

4. Briney, Amanda. (2017). "Low and High Pressure." ThoughtCo, 3 March.

5. Carlton, E.J., Eisenberg, J.N.S., Goldstick, J., Cevallos, W., Trostle, J., Levy, K., (2014). Heavy rainfall events and diarrhea incidence: the role of social and environmental factors. Am. J. Epidemiol. 179, 344–352.

6. Chou, W. C., Wu, J. L., Wang, Y. C., Huang, H., Sung, F. C. & Chuang, C. Y. (2010). Modeling the impact of climate variability on diarrhoea-associated diseases in Taiwan (1996-2007). *Science of the Total Environment*, *409*(1): 43–51. doi:10.1016/j.scitotenv.2010.09.001

7. Cullen E. (2009). The impact of climate change on the future incidence of specified Foodborne diseases in Ireland. Epidemiology. 60(6):S227–8.

8. Department of Statistics Malaysia. (2016). *Population quick info*.

9. El-fadel, M., Ghanimeh, S., Maroun, R. & Alameddine, I. (2012). Science of the Total Environment Climate change and temperature rise : Implications on food- and water-borne diseases. *Science of the Total Environment 437*: 15–21.

10. ESR. (2015). Bacillus Cereus (August), 1–4.

11. Fauziah Mohd Nor. (2015). Prevention & Control of Communicable Diseases During and After The Flood ; Kelantan Experience (October).

12. Health, M. of. (2001). Salmonella typhi. *Ministry of Health*, (May), 1–3.

13. IPCC. 2014. Climate Change 2014 Synthesis Report Summary Chapter for Policymakers. *Ipcc*, 31.

14. IPCC. 2014. Fifth Assessment Report. What's in it for Southeast Asia? Executive Summary. Climate and Development Knowledge Network (CDKN)

15. Ghazani, M., FitzGerald, G., Hu, W., Toloo, G. S., & Xu, Z. (2018). Temperature variability and gastrointestinal infections: a review of impacts and future perspectives. *International journal of environmental research and public health, 15*(4), 766.

16. Kendrovski, V. & Gjorgjev, D. (2012). Climate Change: Implication for Food-Borne Diseases (Salmonella and Food Poisoning Among Humans in R. Macedonia). *Structure and Function of Food Engineering*, 414.

17. Lin, S., Sun, M., Fitzgerald, E., & Hwang, S. A. (2016). Did summer weather factors affect gastrointestinal infection hospitalizations in New York State?. *Science of The Total Environment*, *550*, 38-44.

18. Liu, Z., Liu, Y., Zhang, Y., Lao, J., Zhang, J., Wang, H., & Jiang, B. (2019). Effect of ambient temperature and its effect modifiers on bacillary dysentery in Jinan, China. *Science of The Total Environment*, *650*, 2980-2986.

19. Hassan, N.A, Hashim, J.H., Wan Puteh S.E.,Wan Mahiyuddin, W.R., , Faisal, M.S. (2020). The Impacts of Climate Variability on Cholera Cases in Malaysia. *International Medical Journal Malaysia,*1: 19-27

20. Hofstra, N. (2011). Quantifying the impact of climate change on enteric waterborne pathogen concentrations in surface water. *Current Opinion in Environmental Sustainability*, *3*(6), 471–479.

21. Kim, J. G. (2020). Influence of Climate Factors on the Occurrence of Pathogenic Escherichia coli Food Poisoning in Korea. *Journal of Environmental Health Sciences*, *46*(3), 353-358.

22. Mahirah Kamaludin , Khalid Abdul Rahim, A. R. and M. R. Y. (2013). Improvements in domestic water services in kelantan: are people willing to pay? *Science, Sustainability Volume, Management Umt, Penerbit Studies, Environmental Serdang, U P M*, *8*(2), 197–206.

23. Malaysian Meteorological Department. (2009). *Climate Change Scenarios for Malaysia 2001 - 2099*. Malaysian Meteorological Department.

24. McIver, L.; Hashizume, M.; Kim, H.; Honda, Y.; Pretrick, M.; Iddings, S.; Pavlin, B. Assessment of climate-sensitive infectious diseases in the Federated States of Micronesia. *Trop. Med. Health* **2015**, *43*, 29–40

25. Md. Wahid Murad, Md. Mahadi Hasan, Md. Saiful Islam & Md. Mahmudul Alam. (2014). Socio-Economic Profile Of The Low Income And Poor Communities In Kuala Lumpur City , Malaysia. *International Journal of Ethnics in Social Sciences*, *2*(1), 1–18.

26. Meftahuddin T.(2002). Review of the trends and causes of food borne outbreaks in Malaysia from 1988 to 1997. *Med J Malaysia*. 2002 :57(1):70-9.

27. Ministry of Health (MOH). (2017). *Case definition for infectious diseases in Malaysia.* 3rd Edition. Malaysia.

28. MOH. (2016).Kenyataan Akhbar KPK 28 April 2016: Kejadian Keracunan Makanan di Sekolah Seluruh Malaysia

29. MNRE.(2015). Biennial Update Report to the UNFCCC. Putrajaya, Malaysia.

30. Morral, C., Martínez Solanas, È., Villanueva Belmonte, C., & Basagaña Flores, X. (2018). Weather and gastrointestinal disease in Spain: A retrospective time series regression study.

31. Onozuka, D.; Hashizume, M. Weather variability and paediatric infectious gastroenteritis. *Epidemiol. Infect.* **2011**, *139*, 1369–1378.

32. Othman. (2015). Quantification of Pathogenic Bacteria in Flood Water in Malaysia. Fifth International Conference on Geotechnique, Construction Materials and Environment, Osaka, Japan.

33. Salleh, W. A. H. I. D. A., Lani, M. N., Abdullah, W. W., Chilek, T. Z. T., & Hassan, Z. A. I. T. O. N. (2017). A review on incidences of foodborne diseases and interventions for a better national food safety system in Malaysia. *Malaysian Applied Biology*, *46*(3), 1-7.

34. Soon, J. M., Singh, H. & Baines, R. (2011). Foodborne diseases in Malaysia: A review. *Food Control*, *22*(6), 823–830.

35. Sharifa Ezat, W. P., Netty, D. & Sangaran, G. (2013). Paper review of factors, surveillance and burden of food borne disease outbreak in Malaysia. *Malaysian Journal of Public Health Medicine* 13(2): 1–7.

36. Trenberth. (2011). Changes in rainfall with climate change. *Clim Res* 47: 123–138

37. Valík, A. M. and Ľ. (2012). World ' s largest Science , Technology & Medicine Open Access book publisher Staphylococcus aureus : Characterisation and Quantitative Growth Description in Milk and Artisanal Raw Milk Cheese Production. *Structure and Function on Food Engineering*.

38. Wang, P., Goggins, W. B., & Chan, E. Y. (2018). Associations of Salmonella hospitalizations with ambient temperature, humidity and rainfall in Hong Kong. *Environment international*, *120*, 223-230.

39. WHO. (2015). Boil water. Geneva: World Health Organization.

40. WHO. (2017). Foodborne disease outbreaks: Guidelines for investigation and control. Geneva: World Health Organization.

41. Wong, C. L., Yusop, Z., & Ismail, T. (2018). Trend of daily rainfall and temperature in Peninsular Malaysia based on gridded data set. *International Journal*, *14*(44), 65-72.

42. Zhou, X., Zhou, Y., Chen, R., Ma, W., Deng, H. & Kan, H. (2013). High Temperature as a Risk Factor for Infectious Diarrhoea in Shanghai, China. *Journal of Epidemiology*, *23*(6), 418–423.

43. Zin, T., SabaiAung, T., Saupin, S., Myint, T., Daw Khin, S. N., Aung, M. S. & Shamsul, B. S. (2015). Influencing factors for cholera and diarrhoea: Water sanitation and

hygiene in impoverished rural villages of Beluran district, Sabah Malaysia. *Malaysian Journal of Public Health Medicine, 15*(1), 30–40.

Figure 1

Click here to access/download;Figure;Figure 1 Mean Annual incidence rate per 100,000 population.jpg

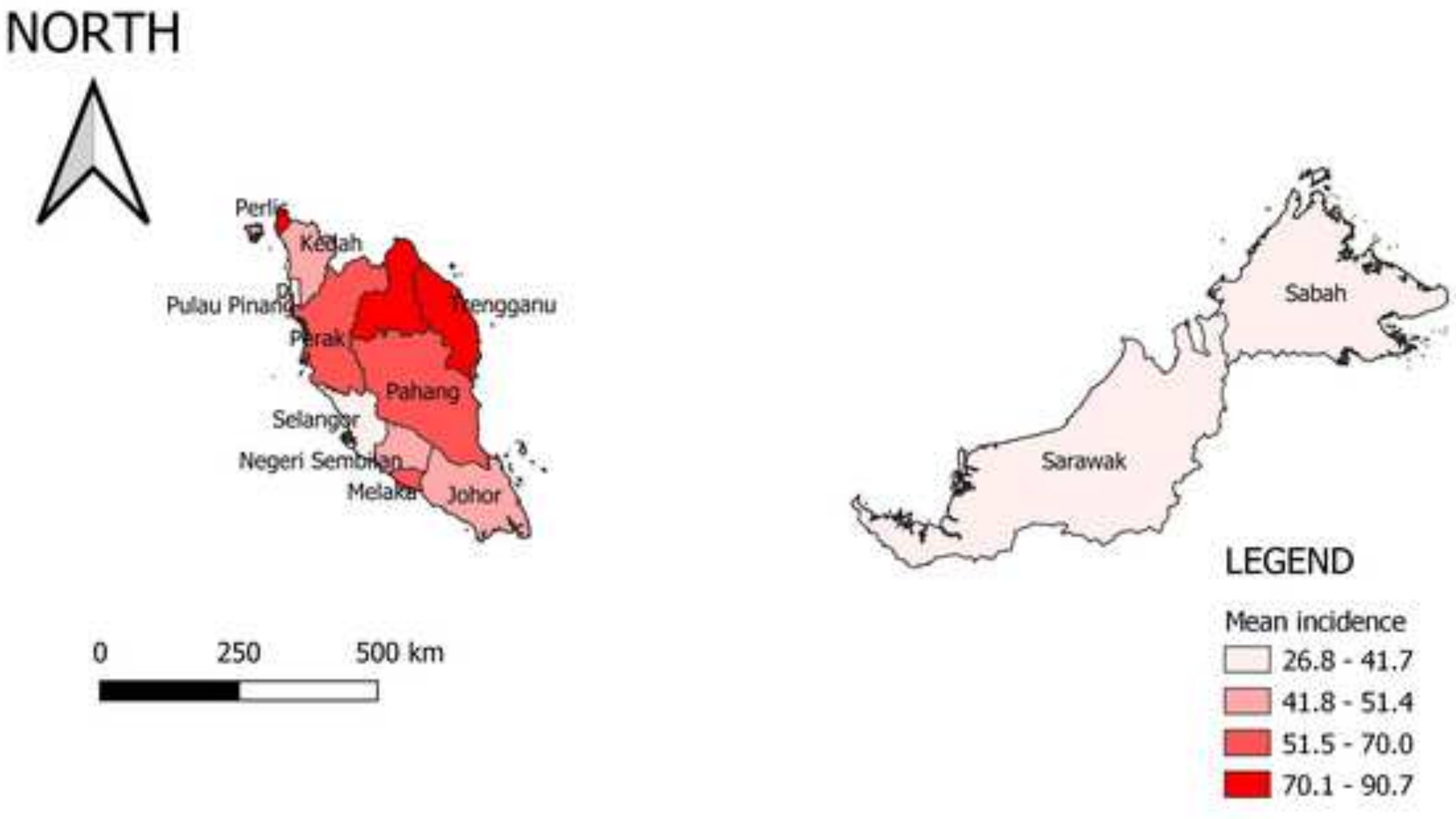

Figure 1: Mean annual incidence rate of food poisoning per 100,000 population

Figure 2

Click here to access/download;Figure;Figure 2 Maximum annual Incidence rate of food poisoning.jpg

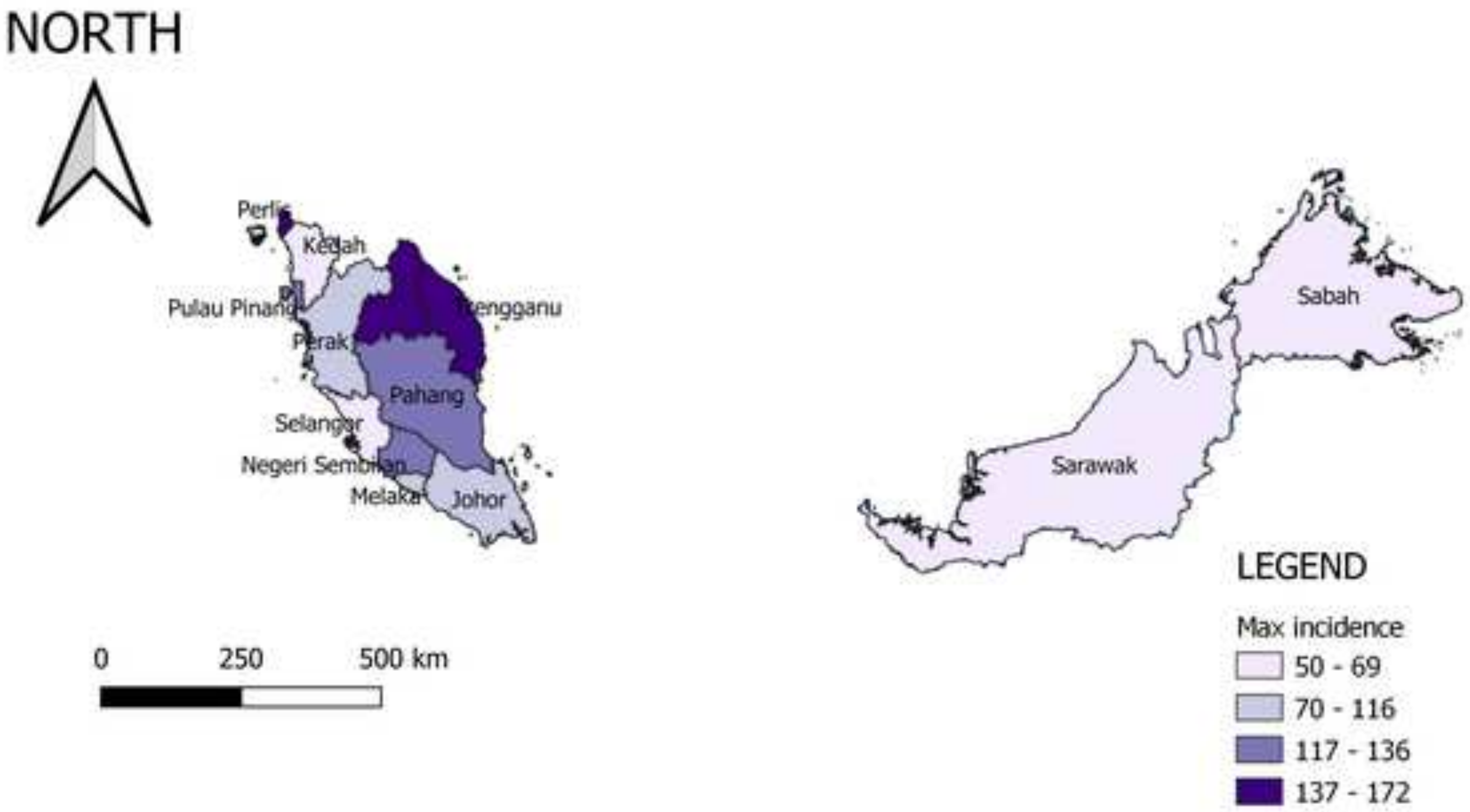

Figure 2: Maximum annual incidence rate of food poisoning per 100,000 population

Figure 3

Click here to access/download;Figure;Figure 3 Mean monthly temperature.jpg

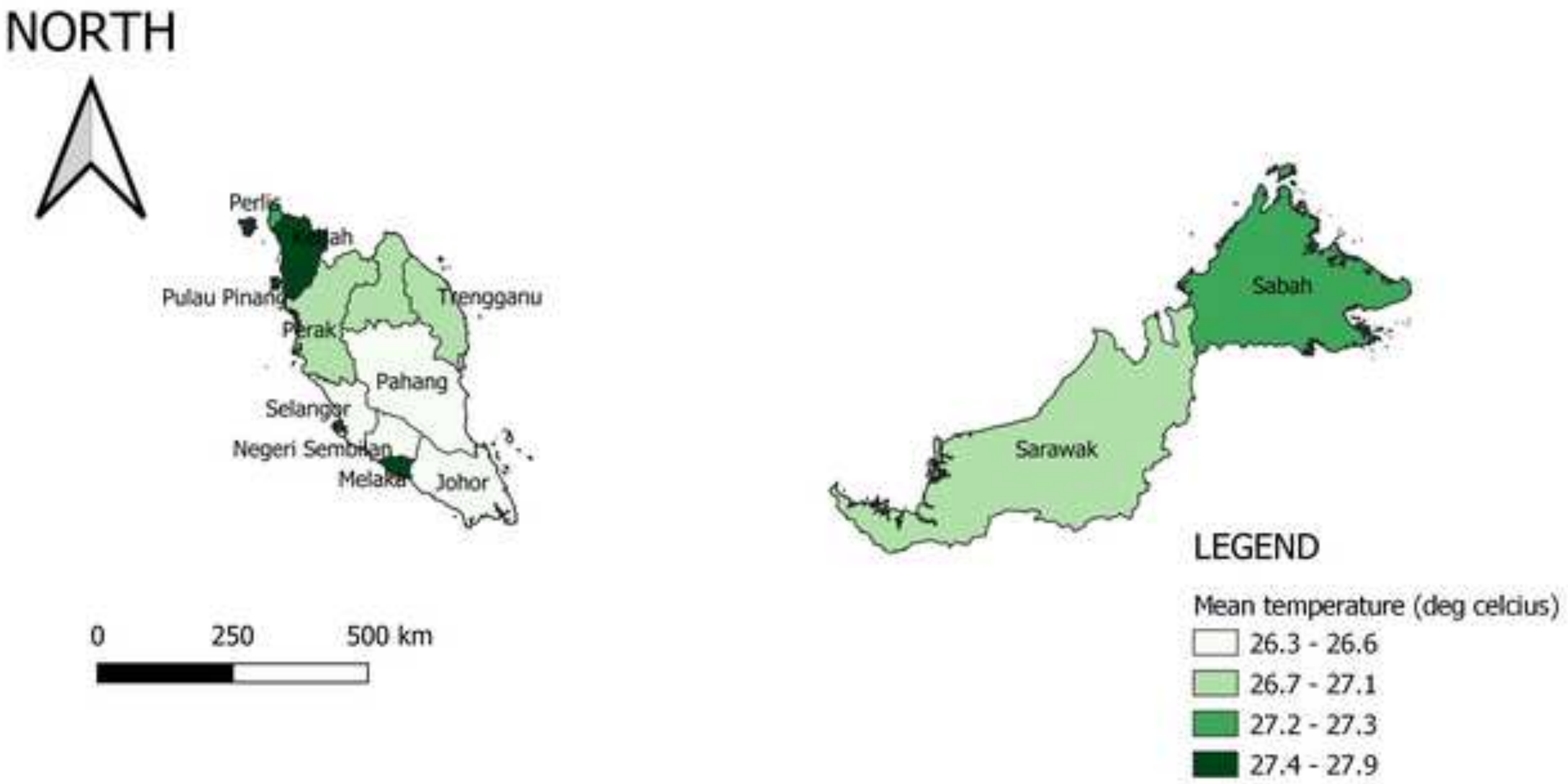

Figure 3: Mean monthly temperature

Figure 4

Click here to access/download;Figure;Figure 4 Maximum mean monthly temperature.jpg

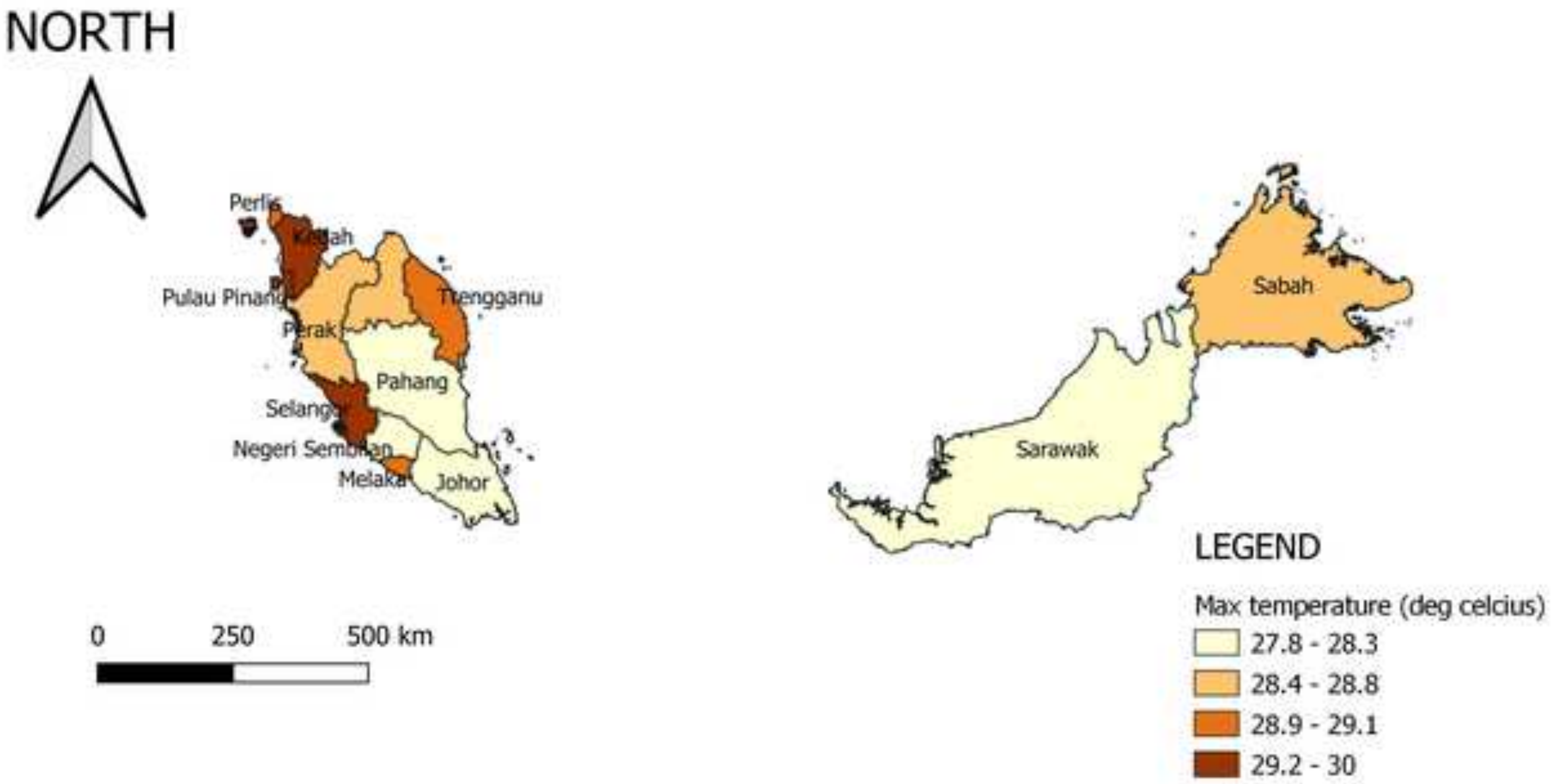

Figure 4: Maximum mean monthly temperature

Figure 5

Click here to access/download;Figure;Figure 5 Mean monthly rainfall.jpg

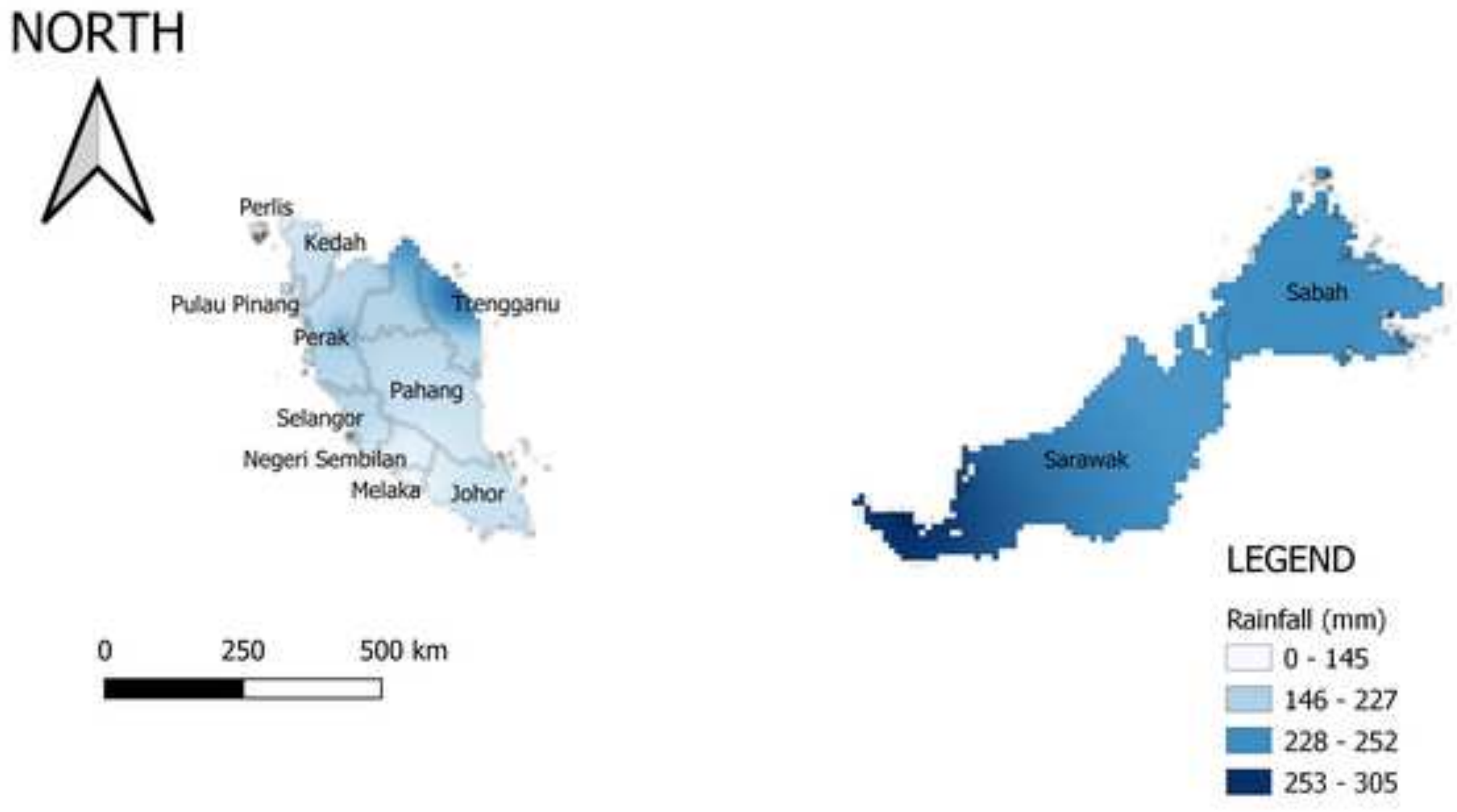

Figure 5: Mean monthly rainfall

Click here to access/download;Figure;Figure 6 Maximum mean monthly rainfall.jpg

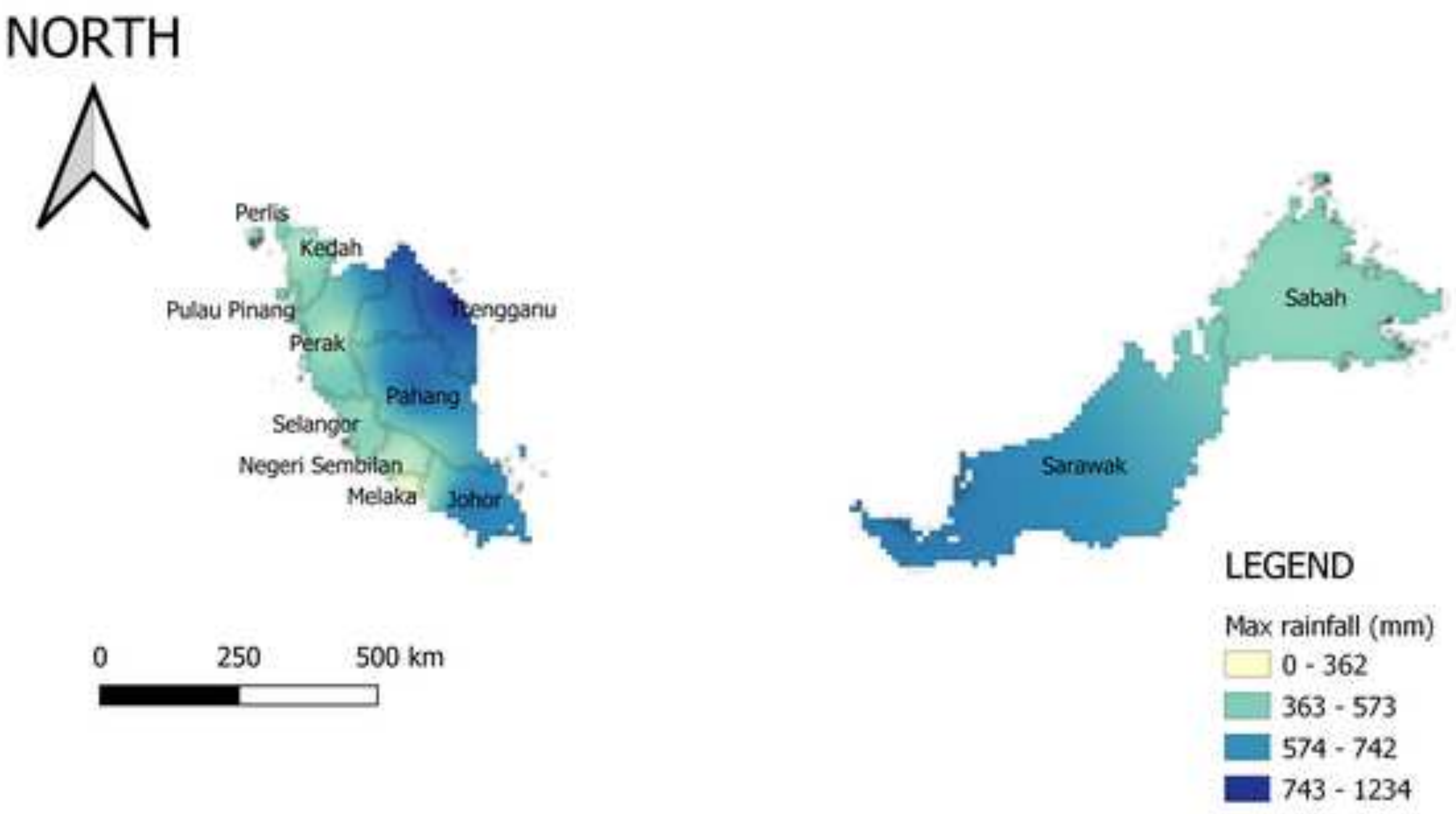

Figure 6: Maximum mean monthly rainfall

Figure 7

Click here to access/download;Figure;Figure 7 Food poisoning in Selangor.jpg

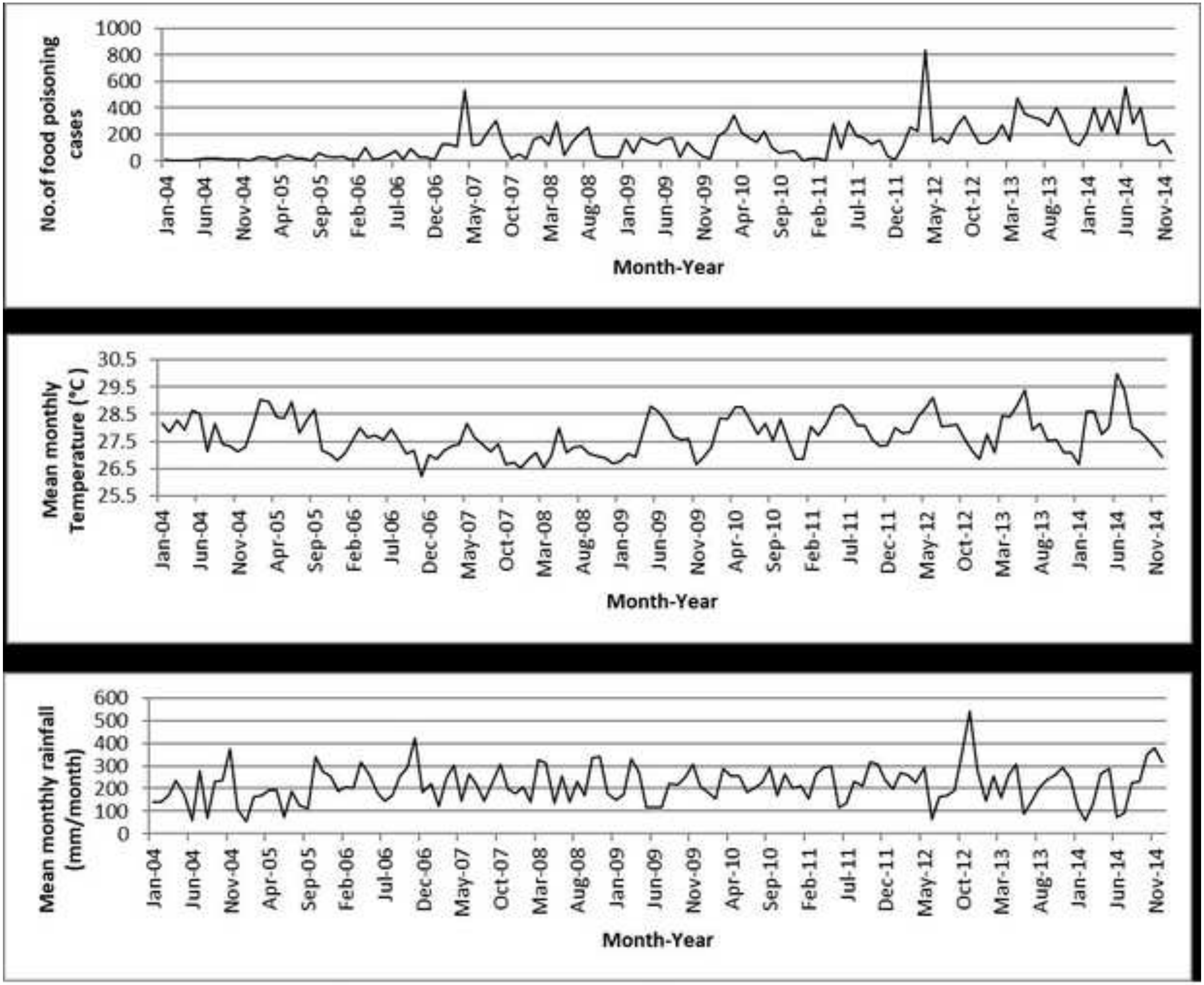

Figure 7: Food poisoning and climate data in Selangor

Figure 8

Click here to access/download;Figure;Figure 8 Food poisoning in Kelantan.jpg

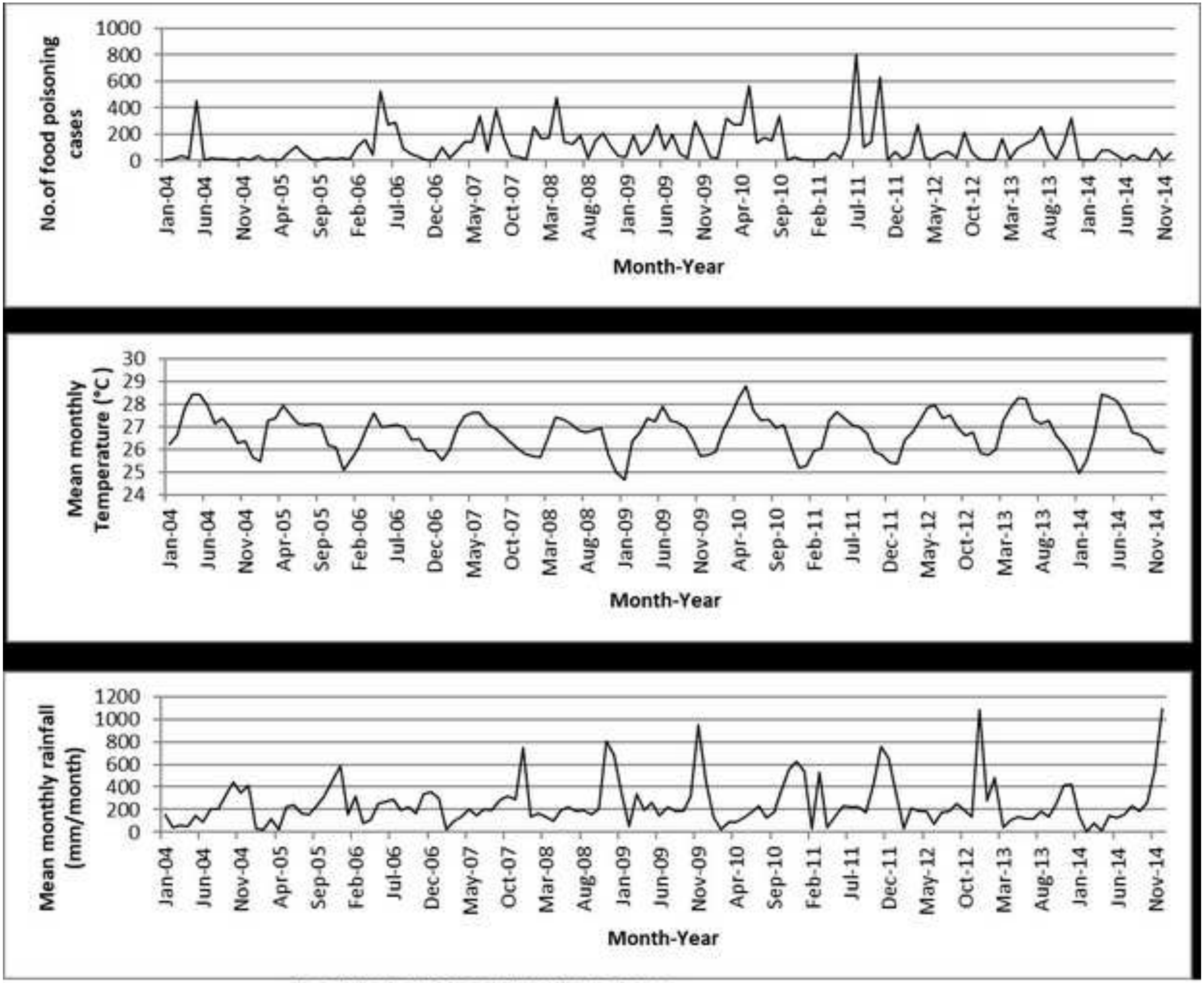

Figure 8: Food poisoning and climate data in Kelantan

Figure 9

Click here to access/download;Figure;Figure 9 Food Poisoning in Melaka.jpg

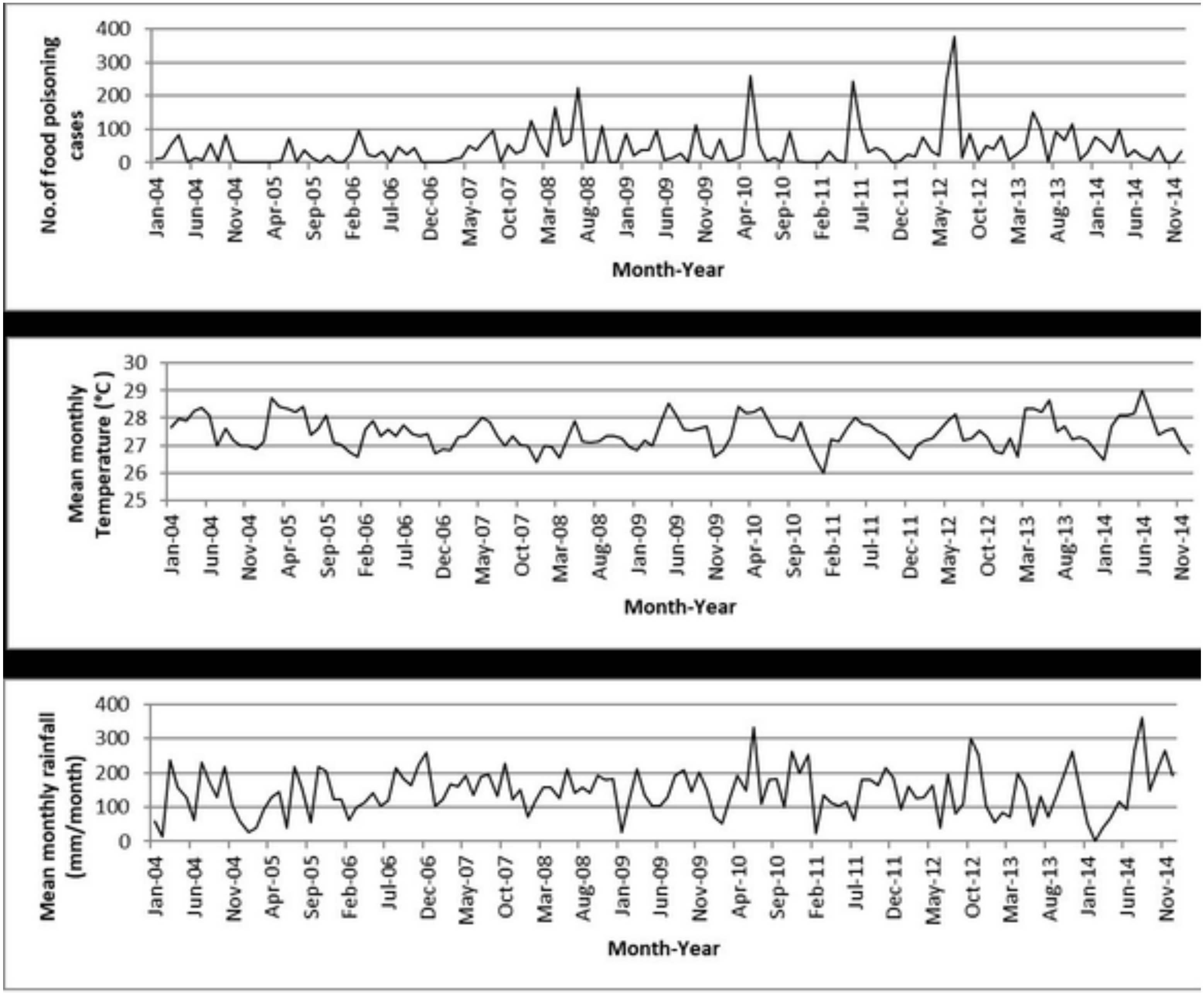

Figure 9 : Food poisoning and climate data in Melaka

Click here to access/download;Figure;Figure 10 Food Poisoning in Sabah.jpg

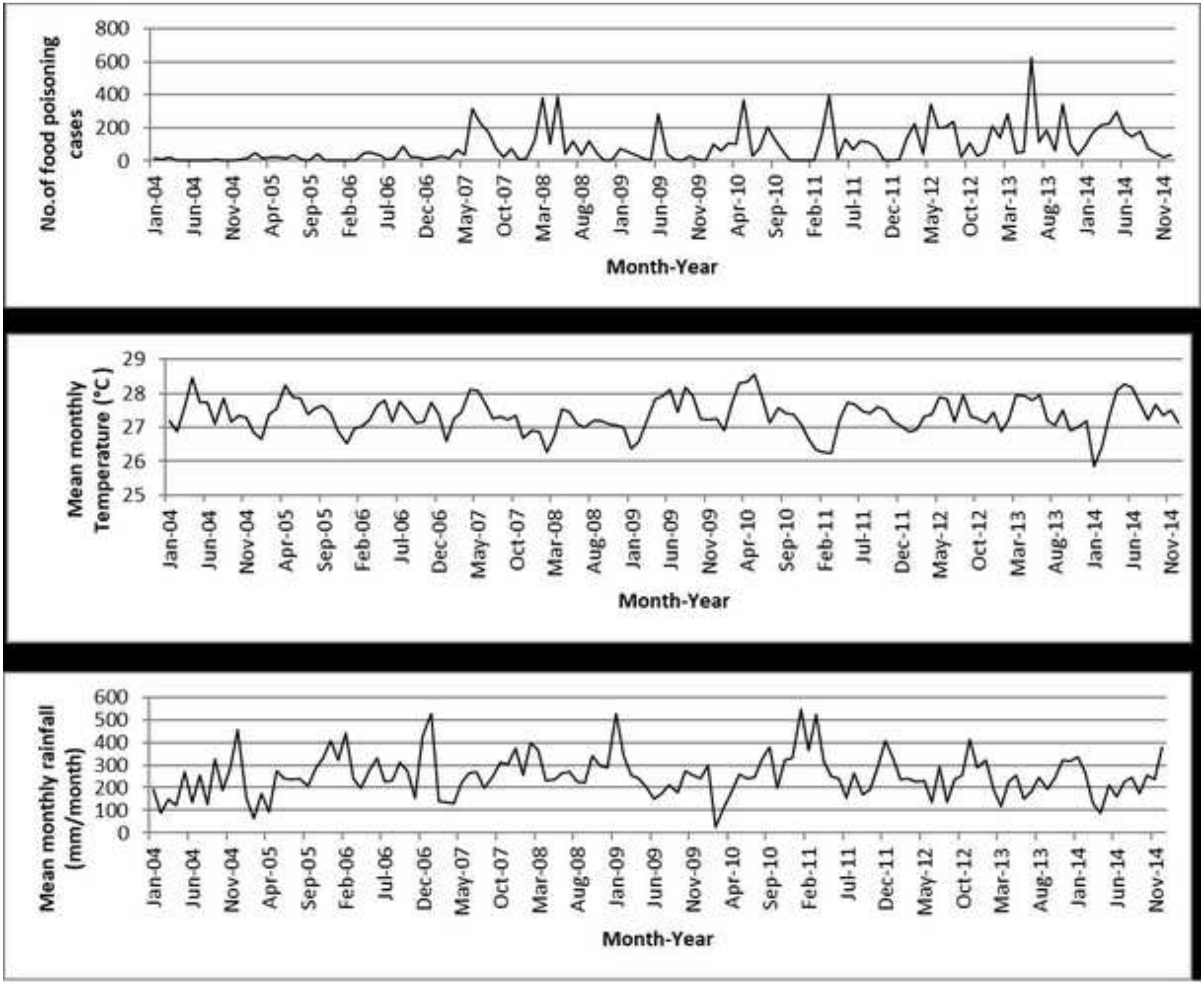

Figure 10 : Food poisoning and climate data in Sabah

Figure 11

Click here to access/download;Figure;Figure 11 Food Poisoning in Terengganu.jpg

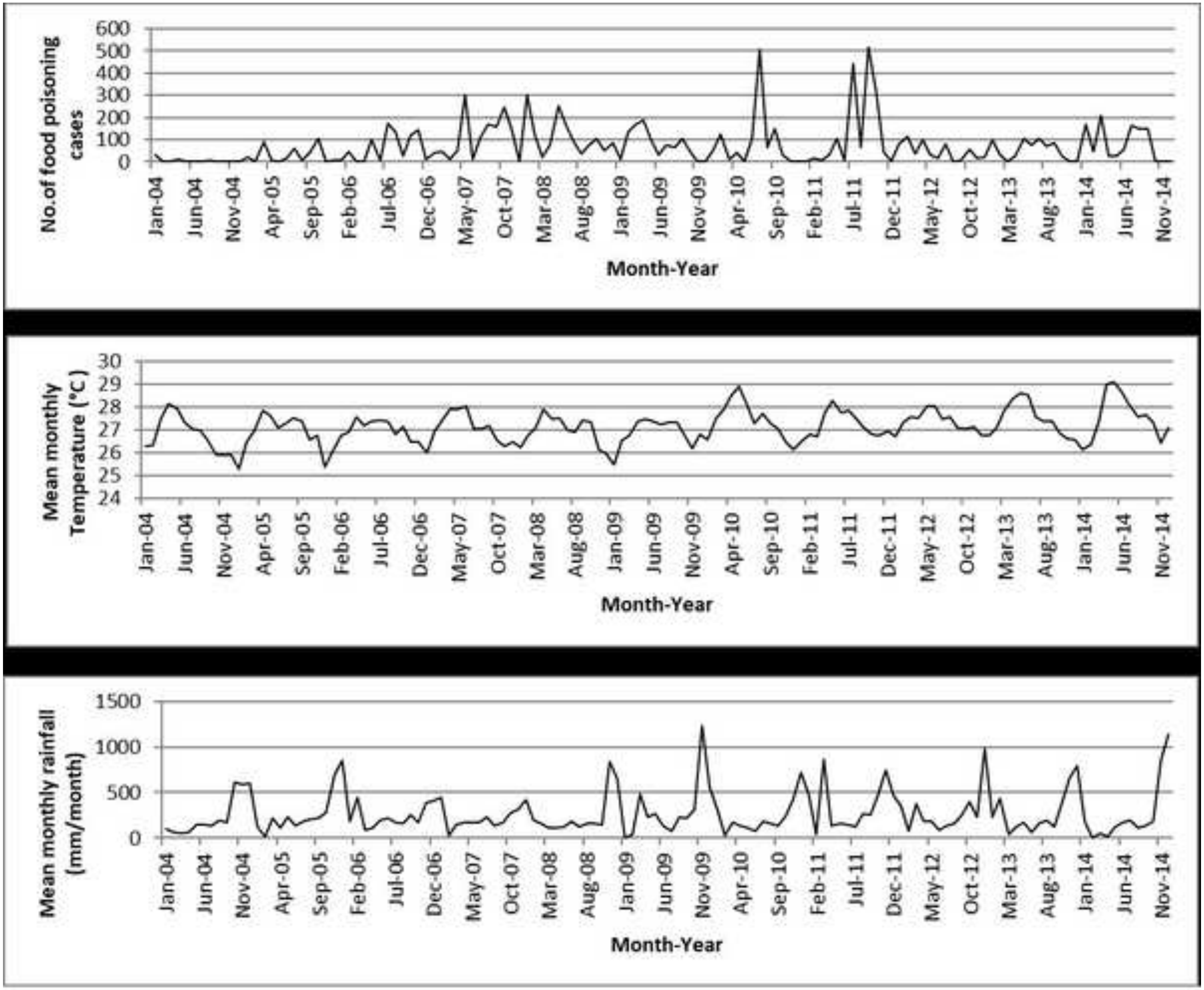

Figure 11 : Food poisoning and climate data in Terengganu

Table 1 Sum of food poisoning incidences in Malaysia from 2004 to 2014.

**Demographic Data**	**Food Poisoning Incidence Proportion (per 100 000 population)**
**Gender**	
Male	249 cases
Female	313 cases
**Race**	
Malay	438 cases
Non-Malay	122 cases
**Age**	
Mean±SD	20±18 years old
0-12 years old	174 cases
13-35 years old	331 cases
36-58 years old	27 cases
>59 years old	5 cases
**Nationality**	
Malaysian	551 cases
Non-Malaysian	10 cases
**Death**	2 cases (per 1 000 000 population)

Table 2 Distribution of annual incidence rates of food poisoning cases and climate in Malaysia from 2004-2014.

	Annual incidence rates from 2004-2014 (per 100,000 population)					Climate from 2004-2014			
**States**	**Min**	**Mean**	**Max**	**High incidence rates**	**Low incidence rates**	**High Temperature**	**Low Temperature**	**High Rainfall**	**Low Rainfall**
Johor	13.13	51.39	97.21	May to June	November to December	May and June	December and January	December and January	February and June
Melaka	21.67	67.22	113.64	April to July	November to December	May and June	December and January	November and December	January and February
Negeri Sembilan	5.99	45.42	135.52	June to July	December	April and May	December and January	October and November	January and February
Selangor	2.80	26.78	50.32	April to June	November to December	June and July	November and December	October and November	January and February
Pahang	38.03	70.03	132.80	July to August	November to December	May and June	December and January	November and December	February and June
Terengganu	6.62	90.69	160.55	April, May, July	November to December	April and May	December and January	November and December	February and March
Kelantan	28.96	90.05	155.89	April to July	December to January	April and May	December and January	November and December	February and April
Perak	14.08	58.65	116.04	January, July to August	November to December	May and June	December and January	October and November	June and July
Penang	8.07	41.72	136.14	January, September to October	December	May and June	November and December	August, September, October	December, January, February
Kedah	16.45	45.79	68.95	February to April, September to October	November to December	Mac to May	November and December	August, September, October	January and February
Perlis	8.68	82.88	172.30	February to April, September to November	December	Mac to May	December and January	March, November, and December	January and February
Sabah	3.15	36.89	58.11	March to June	November to December	April and May	January and February	December and January	February and April
Sarawak	9.81	38.05	58.65	April to July	November to December	June and July	December and January	December and January	February, June and August

Table 3 Correlations between meteorological factors and food poisoning at lag 0 month (model 1) and Lag 1 month (model 2)

	Model 1				Model 2			
**Coefficient**	**Model Value**	**Confidence Interval**	**Exp(B)**	**P-value**	**Model Value**	**Confidence Interval**	**Exp(B)**	**P-value**
**Selangor****								
Temperature	0.212	1.033-1.479	1.236	0.020*	0.031	0.865-1.230	1.032	0.730
Rainfall	0.001	0.999 to 1.003	1.001	0.264	-0.001	0.998 to 1.002	1.000	0.991
Seasonality	0.109	1.039 to 1.198	1.115	0.003*	0.084	1.015 to 1.167	1.088	0.018*
Trend	-0.117	0.887 to 0.893	0.890	<0.001*	-0.116	0.887 to 0.894	0.891	<0.001*
**Kelantan* ***								
Temperature	0.396	1.129 to 1.958	1.487	0.005*	0.297	1.030 to 1.760	1.346	0.030*
Rainfall	0.001	0.999 to 1.002	1.000	0.533	0.001	0.998 to 1.002	1.000	0.819
Seasonality	-0.012	0.854 to 1.143	0.988	0.874	-0.101	0.778 to 1.050	0.904	0.188
Trend	-0.020	0.975 to 0.985	0.980	<0.001*	-0.020	0.976 to 0.985	0.980	<0.001*
**Melaka****								
Temperature	0.389	1.046 to 2.080	1.475	0.027*	0.152	0.826 to 1.640	1.164	0.385
Rainfall	-0.002	0.995 to 1.002	0.998	0.336	0.002	0.999 to 1.005	1.002	0.200
Seasonality	-0.016	0.870 to 1.114	0.984	0.802	-0.069	0.823 to 1.058	0.933	0.281
Trend	-0.007	0.988 to 0.998	0.993	0.007*	-0.007	0.988 to 0.998	0.993	0.010*
**Sabah****								
Temperature	0.555	1.127 to 2.690	1.741	0.012*	0.353	0.932 to 2.173	1.423	0.103
Rainfall	0.002	0.999 to 1.004	1.002	0.191	0.002	1.000 to 1.005	1.002	0.191
Seasonality	-0.037	0.865 to 1.073	0.963	0.497	-0.091	0.820 to 1.017	0.913	0.097
Trend	-0.060	0.938 to 0.946	0.942	<0.001*	-0.060	0.938 to 0.946	0.942	<0.001*

**Terengganu****								
Temperature	-0.244	0.560 to 1.094	0.783	0.152	0.225	0.888 to 1.766	1.252	0.200
Rainfall	-0.002	0.996 to 0.999	0.998	0.034	-0.001	0.998 to 1.001	0.999	0.272
Seasonality	0.113	0.980 to 1.279	1.120	0.095	0.085	0.948 to 1.250	1.089	0.228
Trend	-0.007	0.987 to 0.998	0.993	0.006*	-0.011	0.984 to 0.995	0.989	<0.001*
**Johor****								
Temperature	-0.131	0.546 to 1.343	0.877	0.546	-0.071	0.631 to 1.374	0.931	0.720
Rainfall	-0.002	0.996 to 1.001	0.998	0.133	-0.001	0.997 to 1.001	0.999	0.555
Seasonality	-0.034	0.860 to 1.086	0.967	0.568	-0.054	0.850 to 1.056	0.948	0.331
Trend	-0.034	0.962 to 0.971	0.967	<0.001*	-0.035	0.961 to 0.971	0.966	<0.001*
**Perak****								
Temperature	-0.231	0.577 to 1.092	0.794	0.156	-0.016	0.706 to 1.371	0.984	0.923
Rainfall	-0.001	0.997 to 1.001	0.999	0.484	-0.001	0.996 to 1.001	0.999	0.283
Seasonality	-0.066	0.854 to 1.026	0.936	0.159	0.046	0.949 to 1.155	1.047	0.361
Trend	-0.013	0.983 to 0.992	0.987	<0.001*	-0.014	0.982 to 0.991	0.986	<0.001*
**Pahang**								
Temperature	0.102	0.851 to 1.441	1.107	0.448	-0.168	0.648 to 1.104	0.846	0.217
Rainfall	-0.001	0.996 to 1.002	0.999	0.434	-0.002	0.996 to 1.001	0.998	0.247
Seasonality	-0.077	0.831 to 1.032	0.926	0.162	-0.089	0.823 to 1.017	0.915	0.099
Trend	-0.006	0.990 to 0.999	0.994	0.012*	-0.006	0.990 to 0.999	0.994	0.011*
**Sarawak ****								
Temperature	0.269	0.881 to 1.943	1.309	0.182	-0.049	0.644 to 1.409	0.953	0.808
Rainfall	0.001	0.999 to 1.002	1.001	0.483	-0.001	0.998 to 1.001	1.000	0.824
Seasonality	0.247	1.031 to 1.590	1.280	0.025*	-0.099	0.832 to 0.987	0.906	0.024
Trend	-0.109	0.822 to 0.979	0.897	0.015*	-0.027	0.970 to 0.978	0.974	<0.001*

**Kedah****								
Temperature	-0.222	0.603 to 1.063	0.801	0.124	-0.244	0.607 to 1.010	0.783	0.060
Rainfall	0.001	1.000 to 1.003	1.001	0.077	0.001	0.998 to 1.001	1.000	0.665
Seasonality	-0.151	0.772 to 0.957	0.860	0.006*	-0.022	0.886 to 1.079	0.978	0.656
Trend	-0.018	-0.979 to 0.986	0.982	<0.001*	-0.018	0.979 to 0.986	0.982	<0.001*
**Penang****								
Temperature	-0.302	0.539 to 1.014	0.740	0.061	-0.284	0.562 to 1.009	0.753	0.057
Rainfall	0.001	0.999 to 1.003	1.001	0.392	0.002	1.000 to 1.004	1.002	0.089
Seasonality	-0.045	0.853 to 1.071	0.956	0.437	-0.045	0.852 to 1.072	0.956	0.442
Trend	-0.011	0.984 to 0.993	0.989	<0.001*	-0.012	0.983 to 0.993	0.988	<0.001*
**Negeri Sembilan**								
Temperature	0.110	0.790 to 1.577	1.116	0.534	0.211	0.905 to 1.684	1.235	0.183
Rainfall	-0.002	0.996 to 1.001	0.998	0.198	-0.002	0.996 to 1.001	0.998	0.162
Seasonality	0.051	0.936 to 1.183	1.052	0.395	0.039	0.925 to 1.168	1.040	0.511
Trend	0.009	1.003 to 1.015	1.009	0.003*	0.009	1.003 to 1.014	1.009	0.003*
**Perlis**								
Temperature	0.121	0.742 to 1.717	1.129	0.572	0.054	0.735 to 1.516	1.005	0.771
Rainfall	0.001	0.998 to 1.004	1.001	0.688	0.001	0.997 to 1.003	1.000	0.813
Seasonality	-0.074	0.773 to 1.115	0.929	0.429	-0.091	0.771 to 1.081	0.913	0.289
Trend	0.005	0.998 to 1.012	1.005	0.164	0.005	0.998 to 1.012	1.005	0.166

**Overdispersed Poisson
*Significance at p<0.05

Table 4 Comparison of excess risk at different temperatures and rainfall level

Variable	B	Formula	Exp (B)	Excess Risk
**Selangor Lag 0**				
Temperature increased by 0.25 °C	0.212	Exp (0.25*0.212)	1.0544	5.4%
Temperature increased by 0.5 °C	0.212	Exp(0.5*0.212)	1.111	11.1%
Temperature increased by 0.75 °C	0.212	Exp(0.75*0.212)	1.172	17.2%
Temperature increased by 1 °C	0.212	Exp (0.212)	1.236	23.6%
**Kelantan Lag 0**				
Temperature increased by 0.25 °C	0.396	Exp (0.25*0.396)	1.104	10.4%
Temperature increased by 0.5 °C	0.396	Exp (0.5*0.396)	1.219	21.9%
Temperature increased by 0.75 °C	0.396	Exp(0.75*0.396)	1.345	34.5%
Temperature increased by 1 °C	0.396	Exp (0.396)	1.487	48.7%
**Kelantan Lag 1**				
Temperature increased by 0.25 °C	0.165	Exp (0.25*0.165)	1.042	4.2%
Temperature increased by 0.5 °C	0.165	Exp (0.5*0.165)	1.085	8.5%
Temperature increased by 0.75 °C	0.165	Exp (0.75*0.165)	1.132	13.2%
Temperature increased by 1 °C	0.165	Exp (0.165)	1.179	17.9%
**Melaka Lag 0**				
Temperature increased by 0.25 °C	0.389	Exp (0.25*0.389)	1.102	10.2%
Temperature increased by 0.5 °C	0.389	Exp (0.5*0.389)	1.214	21.4%
Temperature increased by 0.75 °C	0.389	Exp (0.75*0.389)	1.338	33.8%
Temperature increased by 1 °C	0.389	Exp (0.389)	1.475	47.5%
**Sabah Lag 0**				
Temperature increased by 0.25 °C	0.555	Exp (0.25*0.555)	1.148	14.8%

Temperature increased by 0.5 °C	0.555	Exp (0.5*0.555)	1.319	31.9%
Temperature increased by 0.75 °C	0.555	Exp (0.75*0.555)	1.516	51.6%
Temperature increased by 1 °C	0.555	Exp (0.555)	1.741	74.1%
**Terengganu Lag 0**				
Rainfall increased by 1mm	-0.002	Exp (-0.002)	0.998	Decreased risk by 1%
Rainfall increased by 10mm	-0.002	Exp (10*-0.002)	0.980	Decreased risk by 2%
Rainfall increased by 50mm	-0.002	Exp (50*-0.002)	0.905	Decreased risk by 9%

This paper does not have revised manuscript because it was withdrawn due to unavailability of reviewer to review this paper. I have suggested 3 names to review this paper to the editor.

Thank you.
