# Supplementary material for: Investigation of the impacts of climate change and rising temperature on food poisoning cases in Malaysia
Source: PLoS One. 2023 Oct 20;18(10):e0283133. doi: 10.1371/journal.pone.0283133 (PMC10588843; doi:10.1371/journal.pone.0283133)
Supplement: Attachment — Submitted filename: PlosOne_Feb_2022.docx [file pone.0283133.s002.docx]

**Climate Change and Temperature Rise: Implications on Food Poisoning Cases in Malaysia**

**General**

This paper presents findings on an important area of research is in line with the SDGs specific to climate change. Additionally, food poisoning is an important public challenge which has been shown to depend on climate change. The use of retrospective data also provides a large sample size to improve the power of the study without missing the influence of inherent bias of retrospective studies.

**Introduction**

No comments

**Methods**

Authors reported on missing of some demographic characteristics. Authors failed to mention the numbers and proportions that were missing. This should be reported in the methods section for clarity.

In the development and execution of GLM, did authors adjust or control for any variables that may affect incidence of food poisoning. If so, can they add this to the methods section, if not then authors should discuss this as a limitation in the discussion.

**Results**

In table 3, 95% percent confidence intervals were reported, however, none have been used to support the results reported with the p-values. I suggest authors add these the main results section and the results section of the abstract.

**Discussion**

**Authors should discuss the key strength of this study and the inherent limitations of using a retrospective data specific to this study.**

**Conclusion**

No comments
