# Supplementary material for: Investigation of the impacts of climate change and rising temperature on food poisoning cases in Malaysia
Source: PLoS One. 2023 Oct 20;18(10):e0283133. doi: 10.1371/journal.pone.0283133 (PMC10588843; doi:10.1371/journal.pone.0283133)
Supplement: Attachment — Submitted filename: Feedback PLOS ONE.docx [file pone.0283133.s003.docx]

PONE-D-21-31372R2

Climate Change and Temperature Rise: Implications on Food Poisoning Cases in Malaysia


A. Where did the authors obtain the maps, basemaps, shapefiles, map data in Figures 1-6? Please provide any relevant links or copyright information.

Answer: All the data from this link, <https://www.diva-gis.org/gdata,free> spatial data. The shapefiles and map were also obtained from this link.

B. Where did the authors obtain the data used to create the figures in Figures 1-6? Please provide any relevant links or copyright information. Please also specify if these data belong to the authors.

Answer: All these data belong to me as I have obtained approval to use them for my Ph.D. Food Poisoning data was obtained from the Ministry of Health, meteorological data from the Meteorological Department, and population data from the Department of Statistics, Malaysia.


2. Thank you for explaining your data sharing restrictions. Please provide any contact information, access websites, request forms, data set names, etc. that will help interested researchers obtain your same data set. We will help you update your statement accordingly.

1. Morbidity data : Ministry of Health. The application must be registered under the NMRR at <https://nmrr.gov.my/register>. Once approved, we can use the NMRR approval to get the data from the Ministry of Health.
2. Meteorological data: Meteorological data can be brought at this link <https://www.met.gov.my/hubungi-kami/alamat-pejabat/> or email to Pusat Iklim Nasional**,** [klim@met.gov.my](mailto:klim@met.gov.my)
3. Population data is accessible and can be obtained from this link <https://pqi.stats.gov.my/search.php>
