# Supplementary material for: Investigation of the impacts of climate change and rising temperature on food poisoning cases in Malaysia
Source: PLoS One. 2023 Oct 20;18(10):e0283133. doi: 10.1371/journal.pone.0283133 (PMC10588843; doi:10.1371/journal.pone.0283133)
Supplement: Attachment — Submitted filename: feedback 16.1.2023.docx [file pone.0283133.s004.docx]

Manuscript ID: PONE-D-21-31372R2

***Investigation of the impacts of climate change and rising temperature on food poisoning cases in Malaysia***

Our team appreciates the reviewers' time and valuable feedback on our manuscript. We considered their suggestions and were able to address their concerns to produce a greater piece of work**.**

**FIRST REVIEWER**

**Comment 1: Non- Malays are non-citizens I guess, who are the others referring to? I suggest you add all Non-Malays as one group**

Feedback: I have made the changes. In Malaysia, we are a multiracial country consisting of Malay, Chinese, and Indian. They are the majority. Others refer to minorities, such as Iban, Kadazan, Dusun, Dayak, aborigines etc. I can't list them all because they are many. However, I still want to keep Malay, Chinese and Indian because they are the majority in Malaysia and Malaysian. Non-Malaysian refers to immigrants. I addressed this in lines 117-121 (Manuscript highlighted in yellow)

“Native Malays or Bumiputera recorded the highest number with 18.87 million (61.4%), followed by Chinese, Indians, and other races with 6.59 million (21.5%), 1.98 million (6.4%), and 0.27 million (0.9%), respectively. For non-Malaysians or immigrants, there were 3.01 million (9.8%)”

**Comment 2: There is no Table 5 in this manuscript**

Feedback: Amendment has been made from Table 5 to 4 (Lines 297-298).

**Comment 3: From the methodology, the humidity was not a factor in the analysis. you may want to clarify this statement**

Feedback: An amendment has been made, and humidity is removed from the sentence. (Line 380-382)

**THIRD REVIEWER**

Reviewer #3: Climate Change and Temperature Rise: Implications on Food Poisoning Cases in Malaysia

**Comment 1: General**

**This paper presents findings on an important area of research is in line with the SDGs specific to climate change. Additionally, food poisoning is an important public challenge which has been shown to depend on climate change. The use of retrospective data also provides a large sample size to improve the power of the study without missing the influence of inherent bias of retrospective studies.**

Feedback : Thank you

**Introduction
No comments**

**Comment 2: Methods

Authors reported on missing of some demographic characteristics. Authors failed to mention the numbers and proportions that were missing. This should be reported in the methods section for clarity.**

**In the development and execution of GLM, did authors adjust or control for any variables that may affect incidence of food poisoning. If so, can they add this to the methods section, if not then authors should discuss this as a limitation in the discussion.**

Feedback: The missing data is less than 2% of the total cases of 145,543 from 2004-2014. I have added that part in lines 187-189.

I didn’t control for other variables, and therefore, I have added in the limitation part, on lines 390-394,” food poisoning cases can be contributed by multiple factors; while this research only focused on climate factors”.

**Comment 3: Results

In table 3, 95% percent confidence intervals were reported, however, none have been used to support the results reported with the p-values. I suggest authors add these the main results section and the results section of the abstract.**

Feedback: The confidence intervals were added in the abstract and results section (line 33-37 and 252-258) .

**Comment 4: Discussion

Authors should discuss the key strength of this study and the inherent limitations of using a retrospective data specific to this study.**

Feedback: Thank you for your suggestion. The strength and limitations had been added in lines 372-375.

**Conclusion
No comments**

**ACADEMIC EDITOR**

**Comment 1: Format - please check the format of manuscript for the jpurnal eg reference, affiliation etc.**

Feedback : The format has been checked thoroughly from title to references

**Comment 2: Title - not catchy enough. My recommendation: Investigation of impacts of climate change and rising temperature on food poisoning**

Feedback: Thank you for your positive input. The title has been changed to “Investigating the impact of climate change and temperature rise on food poisoning cases in Malaysia”

**Comment 3: Abstract - the methods, approaches, statistical test are not described**

Feedback : Thank you for your comment. The methods, approaches and statistical test were explained in lines 24-28

Monthly food poisoning cases, average monthly meteorological data, and population data from 2004 to 2014 were obtained from the Malaysian Ministry of Health, Malaysian Meteorological Department, and Department of Statistics Malaysia, respectively. Poisson generalised linear models were developed to assess the association between climatic parameters and the number of reported food poisoning cases.

**Comment 4: Line 22 - quantify? or study? investigate?**

Feedback : The term was changed to investigate (line 23)

**Comment 5: Line 26 - quantify? assess?**

Feedback : The term was changed to assess (line 27)

**Comment 6: Line 60-65 - The para discussing on the incidence cases over years. The also need to mentioned that low incidence rate in 1980 compared to recent years are probably due to lack of reported cases or registered cases.**

Feedback : Thank you for the valuable input. It has been added in lines 68-69.

**Comment 7: Line 66-68 - Lack of background of the study. Instead of giving such statement. It is preferable to the author to give details of findings/results from this references 6,7,8,9.**

Feedback : Thanks for your comment. This paragraph has been improved from lines 70-88.

**Comment 8: Line 68 - which country? lack of detail from [6].**

Feedback : Thank you for your comment. I have added extra info and give an example of Lebanon. Line 72-74.

**Comment 9: Fig.5 - what year of rainfall data? averaged from 2004-2014.**

Feedback : Fig 5. Mean monthly rainfall in Malaysia from 2004-2014 (mm/month), line 246

**Comment 10: Fig. 1-6 - the malaysia map are smaller,. I beleive it is impactful if you can crop there rest and merge it into six since all the map are small.**

Feedback : Thank you very much for your suggestion. I have tried to compile everything in a figure, but it was too small and the name of the states was unable to be read. I have also tried to compile two figures in one figure, but the names of the states overlap. Therefore, I would like to humbly request to allow the figure as it was.

**Comment 11: Table 2. different fonts.**

Feedback : Thank you. The size has been changed to Times New Roman, size 12, line 195
